# Operando Evolution of a Hybrid Metallic Alloy Interphase for Reversible Aqueous Zinc Batteries

**DOI:** 10.1002/anie.202416047

**Published:** 2024-12-23

**Authors:** Mingqiang Liu, Kai Yang, Qiming Xie, Nantao Hu, Mingzheng Zhang, Ruwei Chen, Wei Zhang, Jichao Zhang, Feng Shao, Hongzhen He, Roby Soni, Xiaoxia Guo, Jinlong Yang, Guanjie He, Feng Pan, Lu Yao, Thomas S. Miller

**Affiliations:** ^1^ School of Materials Science and Engineering Shanghai Institute of Technology Shanghai 201418 P. R. China; ^2^ Electrochemical Innovation Lab Department of Chemical Engineering University College London London WC1E 7JE UK; ^3^ School of Advanced Materials Peking University Shenzhen Graduate School Shenzhen 518055 P. R. China; ^4^ Advanced Technology Institute Department of Electrical and Electronic Engineering University of Surrey Guildford Surrey GU2 7XH UK; ^5^ Department of Micro/Nano Electronics, Key Laboratory of Thin Film and Microfabrication Technology Ministry of Education) School of Electronics, Information and Electrical Engineering Shanghai Jiao Tong University Shanghai 200240 P. R. China; ^6^ Department of Chemistry University College London London WC1E 7JE UK; ^7^ School of chemistry and chemical engineering Shanghai Jiao Tong University Shanghai 200240 P. R. China; ^8^ Guangdong Research Center for Interfacial Engineering of Functional Materials College of Materials Science and Engineering Shenzhen University Shenzhen 518060 P. R. China

**Keywords:** Zn-ion battery, dendrites, Zn anode protection, metallic alloy interphase, dual-heterometallic protective layer

## Abstract

Aqueous Zn‐ion batteries (AZIBs) are widely acknowledged as viable future energy storage solutions, particularly for low‐cost stationary applications. However, the interfacial instability of zinc anodes represents a major challenge to the commercial potential of Zn‐ion systems, promoting an array of side reactions including spontaneous corrosion, hydrogen evolution, and dendrite growth that destabilize cell performance, lower Coulombic efficiency and ultimately lead to early cell failure. While other commercially relevant battery systems benefit from a spontaneously forming solid electrolyte interphase, no such layer forms in AZIBs. Herein, we have designed and engineered an operando evolved metallic alloy interphase for AZIBs. This interfacial layer is initially deposited in the form of a thin film of Ag and In, but develops in situ to become an intimate mix of an Ag_x_Zn_y_ alloy and metallic indium. Importantly, this dual‐heterometallic layer acts to synergistically regulate the migration of zinc ions through the alloy interphase and enables the dense and planar deposition of Zn, simultaneously overcoming all major drivers of Zn anode degradation. Symmetric and full cells containing this modified metallic zinc anode exhibit stable electrochemical performance, offering high‐capacity retention. Hence, this scalable approach represents a viable route towards the commercial utilization of this energy storage system.

## Graphical abstract

An operando‐evolved metallic alloy interphase is proposed to achieve all‐round protection for Zn metal anodes. This interphase, consisting of Ag_x_Zn_y_ alloy and metallic In, synergistically facilitates the migration of zinc ions through the protective layer and induces their planar deposition. Consequently, the Zn@(Ag−In)||ZnVO full cells demonstrated an excellent capacity retention of 90.2 % over 10,000 cycles.

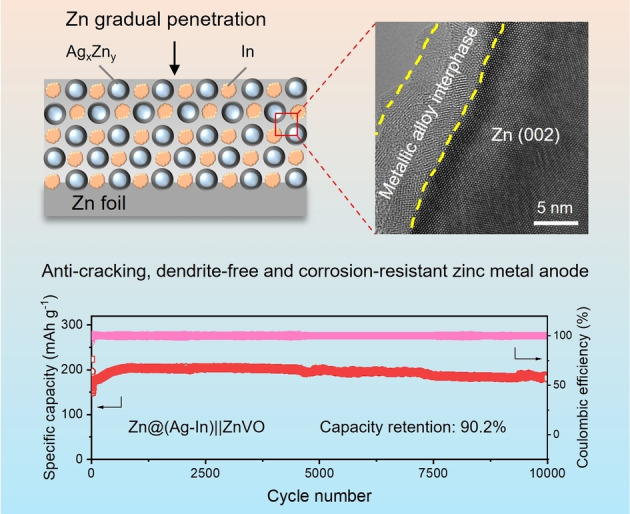

## Introduction

Zinc‐ion batteries with aqueous electrolytes have garnered considerable attention in recent years, as the demand for safer and more cost‐effective energy storage solutions intensifies.^[1]^ Aqueous zinc metal batteries offer several benefits, including the significant natural abundance, high theoretical capacity (820 mAh g^−1^) and a low reduction potential of zinc (−0.762 V vs. standard hydrogen electrode (SHE)), along with the intrinsic safety of water‐based electrolytes.[^2^, ^3^] However, metallic zinc is particularly unstable in weakly acidic salt solutions (e.g., zinc sulfate, zinc chloride), causing undesired passivation, corrosion and the hydrogen evolution reaction (HER) to occur, further exacerbating non‐uniform zinc deposition/dissolution. Hence, if aqueous zinc ion batteries (AZIBs) are to be successful it is necessary to scalably stabilize the metal anode‐electrolyte boundary, while maintaining overall cell performance.^[4]^

In non‐aqueous lithium‐ion batteries the anode‐electrolyte interface is also known to be intrinsically unstable, but stability is endowed by the spontaneous formation of a solid electrolyte interphase (SEI) layer via electrolyte decomposition. Unfortunately, an equivalent stabilizing anode interface layer does not form in traditional AZIBs, and hence, to date, much effort has been dedicated to exploring the formation of engineered SEI layers in aqueous energy storage systems,^[5]^ with strategies either utilizing the generation of an SEI in situ,^[6]^ primarily through electrolyte engineering (Figure S1),[^7^, ^8^, ^9^] or applying an artificial SEI via modification.^[10]^

In contrast to electrolyte‐derived SEIs, artificial SEIs have been shown to be effective in improving the cycle reversibility of zinc metal anodes (ZMAs),[^4^, ^11^] for example, a Zn/indium hydroxide sulfate electrode was reported to provide a stable host structure for buffering volume changes and preventing side reactions.^[12]^ One notable approach involves interface reconstruction via the use of non‐conductive porous materials based on the sieving principle, which promotes a homogeneous zinc ion flux distribution at the metal surface.^[13]^ However, such protective layers can over time become ineffective due to collapse and peeling after extended cycling (Figure S2a). Metal/alloy protective layers have also been shown to act as quasi‐SEI layers, where single‐heterometals such as Ti,^[14]^ Ag[^15^, ^16^] or Zn‐Mn^[17]^ have been shown to help nucleate Zn and regulate the electric field and ion flux on the surface, while Sn,^[18]^ AgZn_3_ ^[19]^ and Ag_x_Zn_y_ ^[20]^ have been shown to induce zinc deposition along the (002) orientation, facilitating uniform zinc nucleation and compact deposition, enhancing overall performance and stability.[^21^, ^22^, ^23^, ^24^, ^25^] However, while these approaches have been proven to be effective in avoiding dendrite growth, it should be noted that here the newly deposited zinc remains at the electrode‐electrolyte boundary, exposed to the weakly acidic electrolyte, meaning Zn corrosion and with parasitic reactions (such as the HER) still inevitably occur (Figure S2b).[^23^, ^26^] Unfortunately, the dense nature of these single‐heterometallic protective layers has to date resulted in high energy barriers and hindered Zn^2+^ ion diffusion, leading to higher charge transfer impedance and polarization, adversely affecting the overall electrochemical performance of the system. To stabilize metallic zinc anodes, it is therefore necessary to develop a truly robust and long‐lived SEI that both controls Zn deposition morphology and totally avoids the exposure of pure metallic Zn to the electrolyte to shield it from detrimental side reactions.

Here, we demonstrate a new design strategy to form an in situ evolved metallic alloy interphase for all‐round protection of ZMAs, which accommodates zinc deposits while inhibiting interfacial side reactions. Importantly, this approach combines the benefits of an artificially deposited SEI with the performance advantages gained from a slowly developing electrolyte‐derived SEI. The dual‐heterometallic interlayer, consisting of Ag_x_Zn_y_ alloy and metallic In, synergistically promotes the migration of zinc ions within the protective layer and induces their deposition along the preferred (002) crystal plane. Concurrently, this metallic alloy interlayer acts as a barrier to prevent zinc corrosion by separating water from the Zn metal. Ultimately, this protective layer ensures the realization of an anti‐cracking, dendrite‐free and corrosion‐resistant ZMA. As a result, the novel ZMA delivers high Coulombic efficiency (CE, 99.8 %) and excellent long‐term cycle life for both zinc symmetric cells (over 8000 cycles) and Zn@(Ag−In)||ZnVO full cells with 90.2 % capacity retention over 10,000 cycles.

## Results and Discussion

### Theory‐Driven Metallic Interphase Design for Zinc Transport and Preferential Deposition

**Figure 1:**
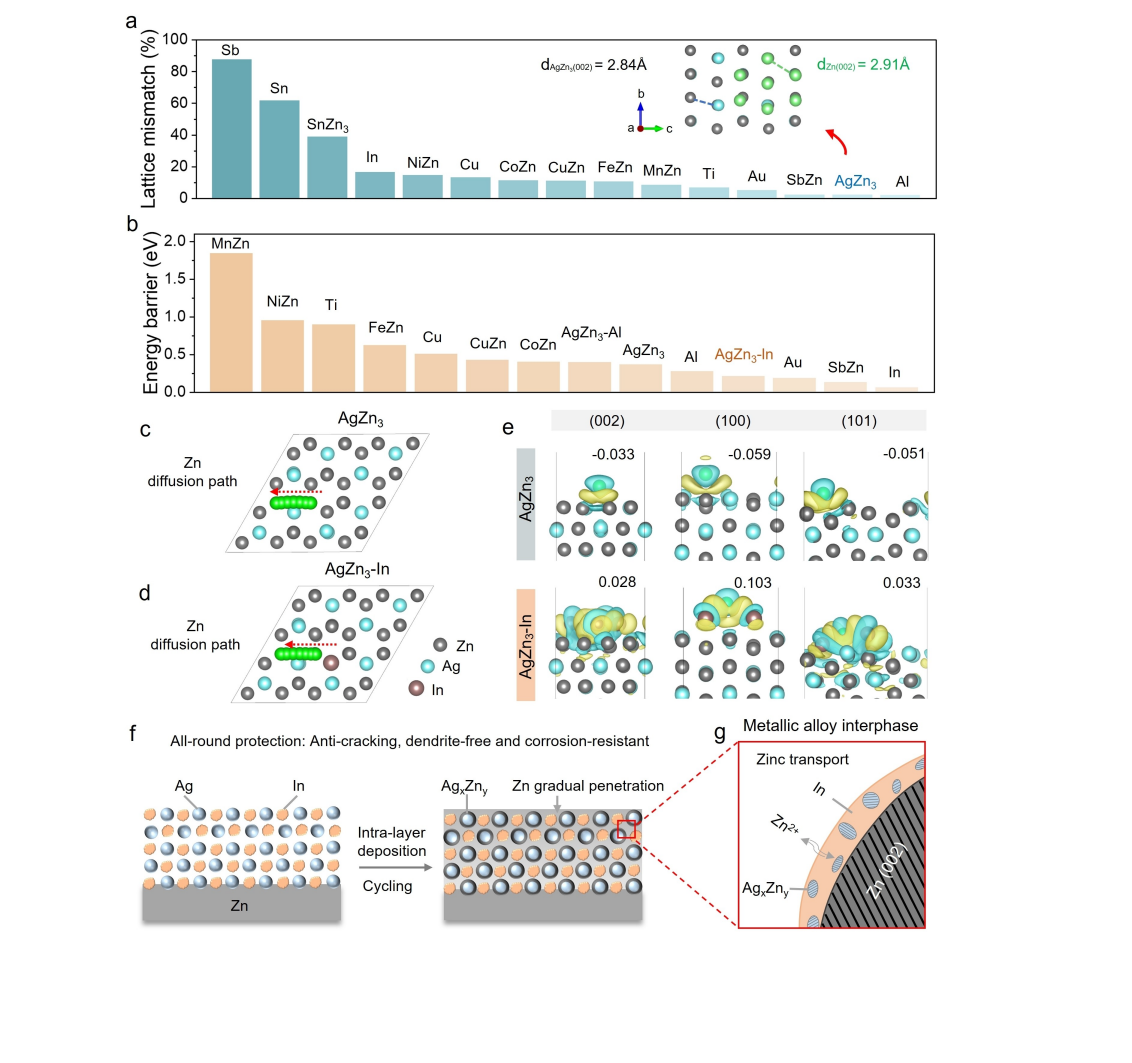
Theory‐driven metallic interphase design for zinc transport and preferential deposition. a) Screening of all the metals and alloys that have been reported in zinc metal protection through lattice mismatch calculation on (002) crystal planes, thw inset shows the atomic arrangement of AgZn_3_ (002) and Zn (002) planes. Atoms are represented by colored spheres: Zn (light green) in Zn (002), Ag (light blue) and Zn (gray) in AgZn_3_ (002). b) Theoretical calculation and screening of zinc diffusion energy barrier through the lattice vacancies of metals with lower lattice mismatch with the Zn (002) plane. c), d) Diffusion models of zinc through AgZn_3_ and AgZn_3_‐In. e) Differential charge density of Zn atoms on (002), (100) and (101) crystal planes of AgZn_3_ and AgZn_3_‐In, respectively. Atoms are represented by colored spheres: Zn (gray), Ag (light blue), In (orange brown), and adsorbed Zn (green). Yellow and blue isosurfaces show electron deficiency and accumulation with an isosurface level of 0.0003 e/Bohr^3^. Bader charges on adsorbed Zn are labeled in each unit. f) Schematic diagram of the evolution of the dual‐heterometallic interlayer during zinc stripping/plating processes. g) Enlarged schematic diagram of the features of the hybrid metallic alloy layer during cycling.

To screen a wide array of materials that could act to promote all‐round zinc anode protection, various theoretical calculations were first performed, including lattice mismatch, migration energy barrier, binding energy, Bader charge and differential charge density simulations to screen promising materials. Lattice mismatch between the substrate and the growth orientation of the depositing crystal, as elucidated using Royer's theory,^[27]^ has been demonstrated to play a key role in facilitating epitaxial growth of crystals, where the depositing material must grow on a substrate with a near‐identical crystalline structure and lattice spacings, with an ideal lattice mismatch of less than 15 %.^[28]^ This concept has also been extended to the oriented deposition of metallic zinc.[^29^, ^30^, ^31^] It is also known that the alignment of the Zn (002) plane parallel to the substrate facilitates a uniform electric field, directing subsequent Zn deposition along this direction to yield a smooth surface (Figure S3), while the reduced chemical activity of the Zn (002) surface diminishes the likelihood of undesired reactions.[^32^, ^33^] Based on these desired properties, a series of metals and alloys were first screened through lattice mismatch calculations on (002) crystal planes (Figure 1a and Figure S4). Among the findings, aluminum and AgZn_3_ showed the lowest lattice mismatch of only 1.84 % and 2.15 % vs the Zn (002) crystal plane respectively, suggesting they would offer an ordered and dense deposition of zinc^,[19]^.^[34]^ Considering the lower redox potential of the Al/Al^3+^ couple (−2.069 V vs. SHE) to Zn, we therefore choose to pursue AgZn_3_ as the main component of our designed protective layer. The inset within Figure 1a shows that the atomic arrangements of the AgZn_3_ (002) and Zn (002) plane almost overlap, indicating a high degree of structural consistency. However, to avoid the deposition of Zn on top of this artificial SEI, it was also necessary to introduce components to promote Zn ion diffusion within and through it. While there is limited data available to describe Zn ion transport in metal layers, In has been shown to offer fast Li‐ion transport through its lattice vacancies (Figure S5), with a Li diffusion energy barrier of only 0.16 eV compared to other reported anode materials (e.g. Ge, Al, Sn).[^35^, ^36^, ^37^, ^38^, ^39^, ^40^, ^41^] To screen the energy barrier for zinc diffusion through the lattice vacancies of metals with a low lattice mismatch with Zn (002), we conducted climbing image nudged elastic band (CI‐NEB) calculations. The results show that In has the lowest zinc diffusion energy barrier of only 0.059 eV (Figure 1b and Figure S6). Hence, in pursuit of a dendrite‐free and corrosion‐resistant metal anode, we proposed a dual‐heterometallic protective layer combining AgZn_3_ with In to synergistically facilitate the migration and deposition of zinc ions. Further calculation results demonstrate that the diffusion energy barrier of Zn in AgZn_3_‐In is significantly lower than in pure AgZn_3_, at 0.21 eV compared to 0.36 eV (Figure S6o). Thus, theoretically zinc ions would migrate more easily along the lattice boundaries and hence pass through this composite alloy layer to reach the Zn metal anode more rapidly (Figure 1d) than through a pure AgZn_3_ lattice (Figure 1c) under an electric field. Next, to investigate the preferred growth orientation of zinc after incorporating In into AgZn_3_, we performed calculations of the binding energy (Figure S7), Bader charge and differential charge density (Figure 1e and Figures S8, 9) of Zn atom adsorption on the low‐index crystal planes of hexagonal Zn, hexagonal AgZn_3_, and AgZn_3_‐In. The binding energy outcomes revealed that the (101) plane possesses the lowest value for hexagonal Zn, which is in agreement with its XRD PDF card (Zn, PDF#04‐0831), where the (101) plane is the main peak. Remarkably, the (002) plane showed the most negative value of −1.40 eV in the AgZn_3_‐In structure, indicating that the inclusion of In markedly enhances the zincophilic ability of the (002) surface, thus guiding zinc deposition preferentially along this plane. The optimized atomic configurations and charge analyses revealed information regarding the adsorption mechanisms. Importantly, while electron loss was observed for AgZn_3_ and Zn, the Bader charges for AgZn_3_‐In surfaces were positive, signifying electron gain. This is consistent with the behavior of Zn atoms on the In (002) plane, suggesting that the interaction with In contributes electrons to the Zn atom.

Analysis of the Ag−In‐Zn ternary phase diagram (Figure S10) shows that a small amount of Zn reacts with Ag to form AgZn_3_, while In does not react with both Ag and Zn at the initial stage.^[42]^ Hence, based on the above theoretical calculations and analysis, we reasonably proposed an initial protective layer consisting of a sputtered Ag and In layer (Figure 1f). During zinc plating/stripping activation cycles, Ag particles progressively form Ag_x_Zn_y_ alloys with zinc atoms, which, in turn, serve as a template to induce the ordered epitaxial deposition of zinc along the (002) crystal plane (Figure 1g). While In remains unreacted, it effectively lowers the migration energy barrier of zinc ions within the interlayer, inducing their transport through the layer. This evolving metallic alloy interlayer is analogous to the formation of an SEI in a lithium‐ion battery, although the layers are structurally, chemically and mechanistically very different. Nonetheless the interfacial layer enables zinc migration and deposition, and concurrently acts as a barrier against corrosion by separating water from the Zn metal, thus achieving all‐round protection for metallic Zn.

### Structural Characterization of the Operando Evolved Metallic Alloy Interphase

**Figure 2:**
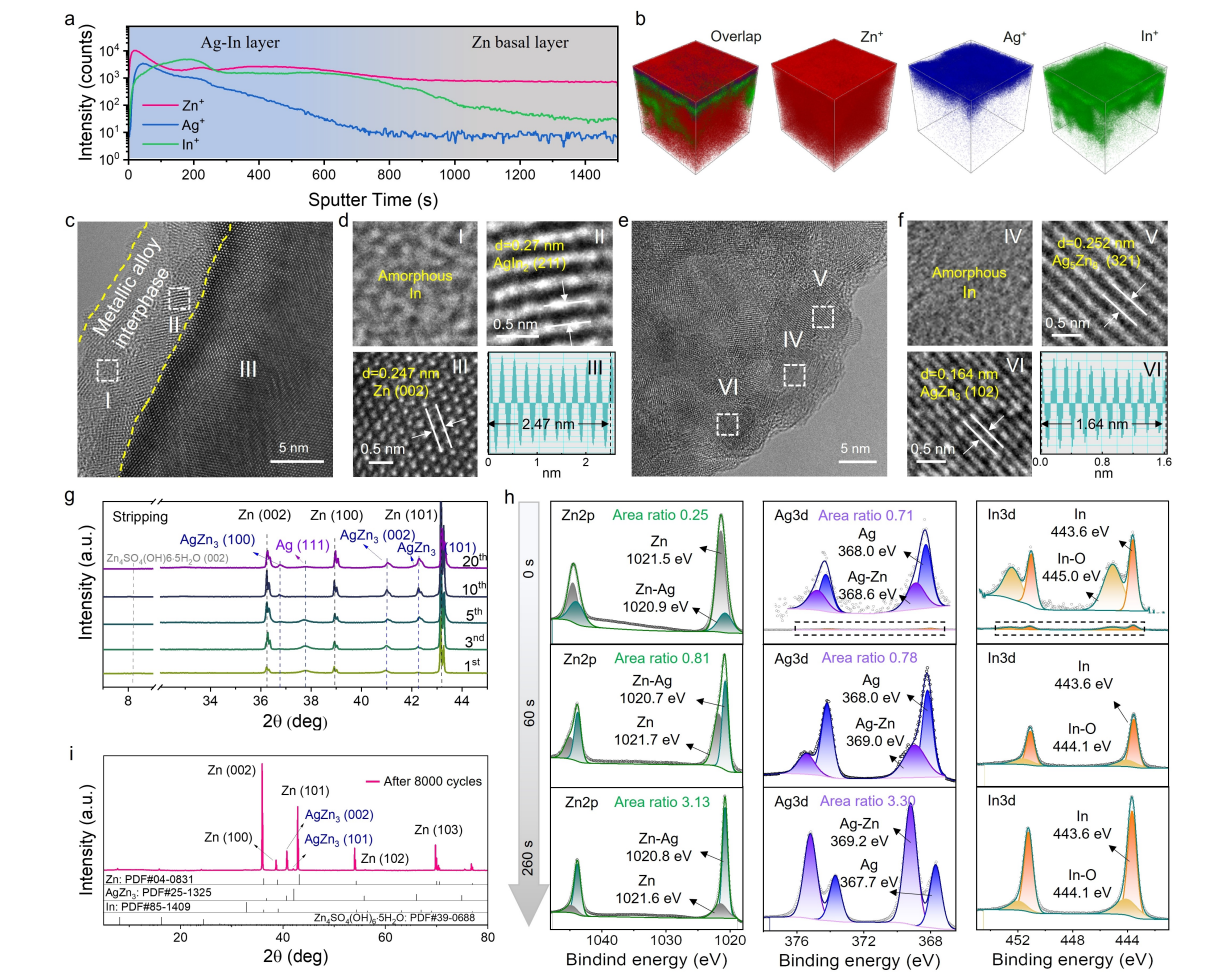
Structural characterization of the operando evolved metallic interlayer. a) Intensity‐time curves and b) spatial distribution of Zn^+^, Ag^+^ and In^+^ on cycled Zn@Ag−In detected by ToF‐SIMS. c), d), e), f) HRTEM images of the cycled Zn@Ag−In electrode. g) Ex situ XRD patterns of Zn@Ag−In electrodes in symmetric cells at different stages of cyclic stripping. h) XPS spectra of Zn2p, Ag3d and In3d for the Zn@Ag−In electrode after 50 cycles with sputtering times of 0 s, 60s and 260s. i) XRD pattern of the cycled Zn@Ag−In electrode in a symmetric battery after 8000 cycles and Zn, AgZn_3_, In and Zn_4_SO_4_(OH)_6_⋅5H_2_O standardized XRD patterns.

Based on the above findings, we modified bare zinc foils by direct current sputtering of silver and indium (experimental details additional data can be found in the supporting information, Figure S11–S17). From this as‐deposited interface layer, it is expected that a true artificial SEI will form during formation cycling (as is expected for a traditional Li‐ion battery SEI), via surface rearrangement and Zn‐ion diffusion within the surface structure. To experimentally verify the migration of zinc into the interlayer, the modified ZMA was cycled in a symmetric cell for 10 cycles (Figure S18). Time‐of‐flight secondary ion mass spectrometry (ToF‐SIMS) analysis of this ‘formed’ anode showed a continuous and uniform distribution of elemental Zn from the surface to the bottom of the cycled electrode (Figures 2a, 2b), demonstrating the successful migration of zinc into the bulk phase of the composite metallic interlayer, which aligns well with the CI‐NEB theoretical calculations (Figure 1b). Additionally, examination of another protected electrode after 20 cycles (Figure S19) using focused ion beam scanning electron microscopy (FIB‐SEM), as shown in Figure S20, revealed through intensity‐distance curves that zinc was also present within the interlayer. This confirms the dynamic nature of this metallic alloy interlayer during formation.

To further explore the surface structure evolution of the Zn@Ag−In electrode during zinc insertion/extraction, protected electrodes were cycled in symmetric cells (Figure S21), and then analyzed using a combination of techniques including high‐resolution transmission electron microscopy (HRTEM), Energy‐dispersive X‐ray spectroscopy (EDS), X‐ray diffraction (XRD) and X‐ray photoelectron spectroscopy (XPS). In Figures 2c,d and Figure S22a, an anode interface is shown, in which bulk Zn appears darker and the composite surface layer is lower contrast. The composite layer consists of both amorphous (In) and crystalline (e.g., AgIn_2_) materials, while the bulk phase is characterized as a single crystal of Zn (002) with a d‐spacing of 0.247 nm. Analysis of another position of the anode interface, shown in Figures 2e,2 f and Figure S22b, revealed that the hybrid metallic alloy interlayer consists of amorphous In and alloys such as AgZn_3_ (102) and Ag_5_Zn_8_ (321), suggesting a gradual alloying process occurs. TEM‐EDS mapping (Figure S23) further shows the uniform distribution of Zn, Ag and In elements on the surface of the cycled electrode. Importantly, the TEM images in Figure S24 corroborate the reproducibility of the interfacial structure formed within the metallic interlayer. Ex situ XRD experiments on the composite metal electrode after electrochemical cycling (Figure 2g and Figures S25, S26) revealed the emergence of small additional peaks at 2θ=36.8°, 41.0° and 42.2°, which can be assigned to AgZn_3_ (PDF#25‐1325), indicating that AgZn_3_ is the primary component of the alloy interphase. Additionally, a distinct peak corresponding to Ag is observed at 37.8°. As the cycle number increases, the peaks attributed to the alloy AgZn_3_ become more pronounced while, simultaneously, there is a noticeable decrease in the intensity of the Ag peak, suggesting the Ag eventually becomes fully alloyed with Zn.

The elemental composition and distribution within the composite alloy layer after cycling were further investigated by XPS. As shown in Figure 2h, Figure S27 and Table S1–3, the presence of Zn−Ag alloys was confirmed by the detection of characteristic peaks at close to 1020.9 eV in the Zn 2p_3/2_ spectrum and 368.6 eV in the Ag 3d_5/2_ spectrum at the surface. Surprisingly, both Zn and the Zn−Ag alloy were identified after ion etching up to 260 s, demonstrating the deep diffusion and deposition of Zn into the bulk phase of the Ag−In layer. Furthermore, the area ratio of alloy‐to‐Zn and alloy‐to‐Ag gradually increased with the depth, implying a gradual penetration of Zn into the composite Ag−In interlayer as the cycle number increases. It is also noteworthy that In remained monolithic except for only a minor portion undergoing oxidation, implying that In is not alloyed with Zn or Ag, which aligns well with the Ag−In‐Zn ternary phase diagram (Figure S10), where In does not alloy with Zn at any ratio.^[42]^ These data demonstrate that the developed interfacial layer consists of both crystalline AgZn_3_ and amorphous In.

XRD patterns of the modified electrode after 8000 cycles (Figure 2i and Figure S28) show a sharp peak at 36.2° 2θ, corresponding to the (002) plane, which emerges as the main peak, consistent with the TEM images presented in Figures 2c‐2 f. Very distinct hexagonal AgZn_3_ peaks can also be found, which is in agreement with the above ex situ XRD patterns, showing that AgZn_3_ represents the main component of alloy layer, further proving the stability of the metallic alloy interphase over exceptionally long period of cycling. It is also worth noting that no distinct peaks could be found below 20° 2θ, indicating that few by‐products formed on the surface of the cycled metal anode, even after extensive long‐term operation. In summary, zinc ions have been shown to successfully migrate through the metallic alloy interphase and deposit along the (002) crystal plane, which is in good agreement with the above theoretical calculations.

### Dendrite Suppression and Anti‐Corrosion Behavior

**Figure 3:**
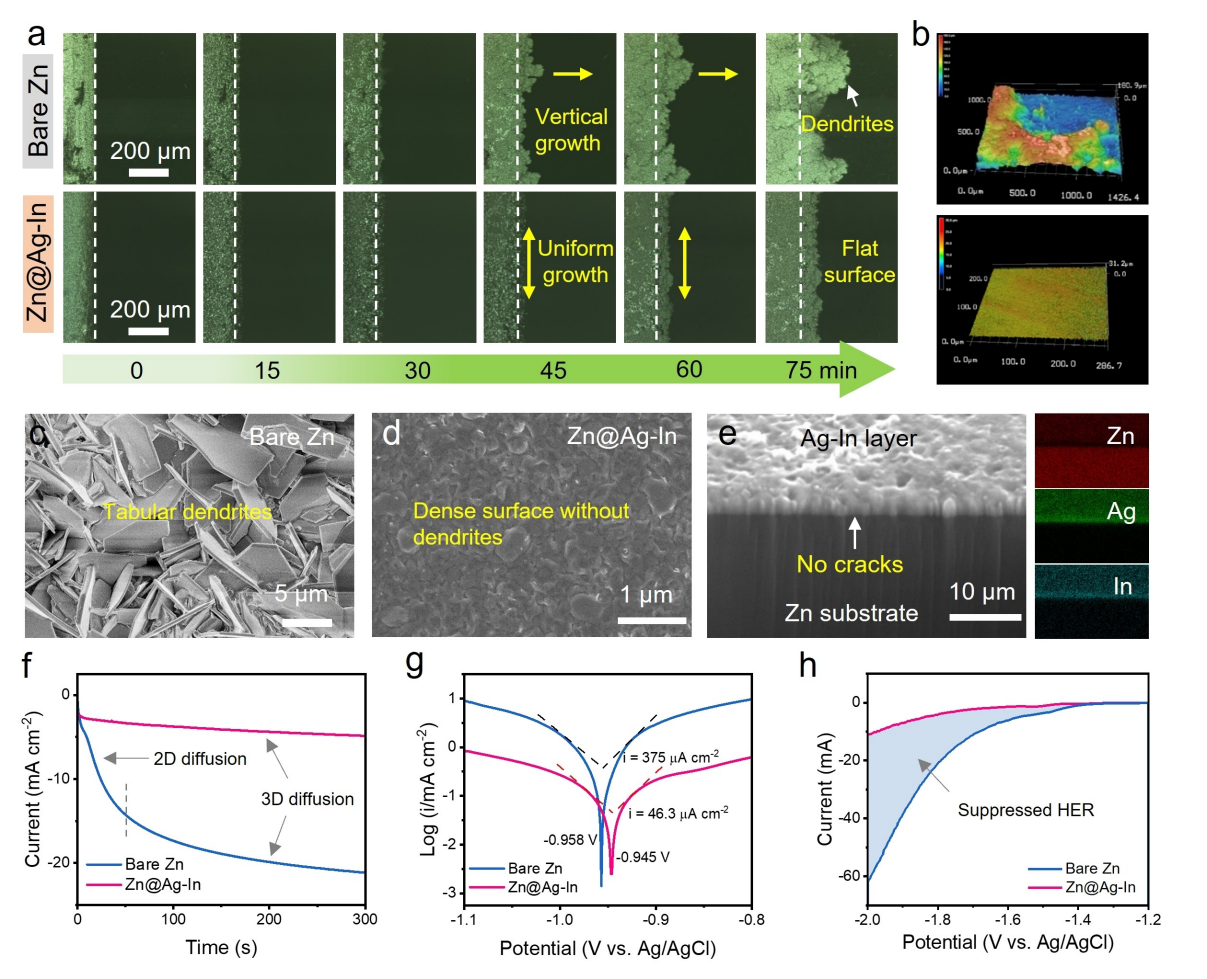
Dendrite suppression and anticorrosion behavior. a) In situ optical LCSM images of Zn electroplating behavior on the two electrodes at 5 mA cm^−2^. b) 3D LCSM images of the two electrodes after 75 minutes deposition. c), d) Top view of SEM images of both electrodes from symmetric cells after 100 cycles. e) Corresponding cross section and elemental distribution of the Zn@Ag−In electrode after cycles. f) Chronoamperometric curves of Zn electrodeposition at −150 mV overpotential. g) Tafel and h) HER plots of both electrodes obtained using a three‐electrode system.

In situ optical laser confocal scanning microscopy (LCSM) was used to monitor the real time surface structural evolution of zinc electroplating on bare Zn foil and Zn@Ag−In electrode. At the initial stage, namely at open circuit potential, the surface of both electrodes was smooth. As time increased, the snapshots of the cross sections at different plating stages became significantly different, as shown in Figure 3a. At a bare Zn anode surface, metal deposited preferentially around the uneven active sites to form large moss‐like blocks, while the surface of the composite metal anode remained uniform and smoother, even after an extended time electrodeposition, which can be attributed to the redistributed and homogeneous reactive sites formed by Ag and In. The top‐down view of the above samples under 3D LCSM (Figure 3b) showed both dendritic protrusions and areas with little deposition for the plated bare foil, whereas the surface of plated modified foil remained flat and smooth. SEM images also showed a stark comparison between bare Zn foil and modified electrode after 100 cycles in symmetric cells (Figures 3c, 3d and Figure S29). Contrary to the rough and dendritic surface of untreated zinc, the cycled Zn@Ag−In electrode showed a flat and smooth surface morphology. All these comparative observations suggest that the metallic protective layer possesses a significant capacity to suppress the formation of dendritic protrusions. Moreover, the corresponding cross‐sectional images show that the metallic interlayer is tightly bonded to the Zn substrate and no gaps are visible, suggesting that the protective layer is unlikely to delaminate during the plating/stripping process (Figure 3e).

The diffusion and deposition behaviors of zinc ions at the two electrode surfaces were then characterized by chronoamperometry (Figure 3f). The rapid increase in current density at the initial stage at the pristine foil can be attributed to 2D diffusion, where ions laterally migrate along the surface for electroreduction at energetically favorable sites. While the flat and plateaued behaviour, sonsistently observed for the protected anode, represents 3D diffusion behavior, suggesting that Zn^2+^ ions adsorbed on the surface undergo local deposition.[^33^, ^43^, ^44^] This phenomenon is largely attributed to the homogeneous electric field and reactive sites provided by the Ag−In layer, facilitating an even electrodeposition process. The corrosion resistance of the Zn anodes was also evaluated by Tafel analysis and HER tests, under three electrodes conditions. A corrosion current density of 375 μA cm^−2^ was measured at the interface of bare foil, however, that dramatically decreased to just 46.3 μA cm^−2^ after protection with the Ag−In layer (Figure 3g). The increase in the HER overpotential further shows the protective layer enhanced the inhibition of H_2_ generation (Figure 3h). These findings collectively demonstrate the superior corrosion resistance offered by the novel anode.

### Identifying Polarization Contributions using Distribution of Relaxation Times Analysis

**Figure 4:**
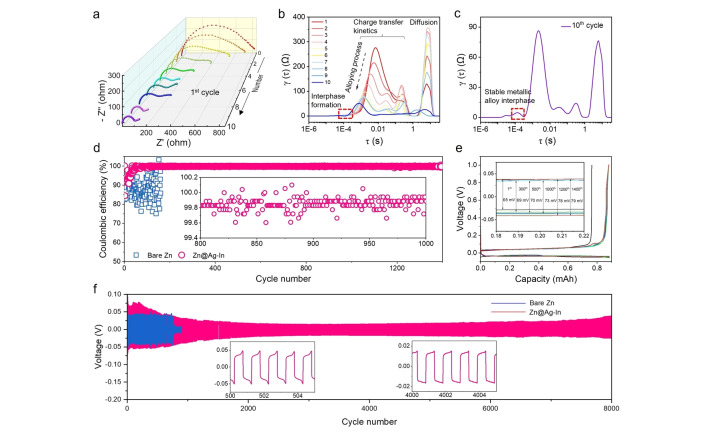
Identifying polarization contributions using DRT analysis. a) Ex situ EIS spectra and b) the corresponding DRT plots of the Zn@Ag−In||Zn@Ag−In symmetric battery at different cycle times within the 1^st^ cycle (EIS was tested every 6 minutes). c) The DRT plot of the Zn@Ag−In||Zn@Ag−In symmetric battery after 10 cycles. Electrochemical performance of half cell with a protected Zn‐metal anode. d) Coulombic efficiency and e) the corresponding polarization curves of Cu||Zn half cells at 1 mA cm^−2^, 0.5 mAh cm^−2^. f) Long‐term cycling performance of symmetric cells with both electrodes at 1 mA cm^−2^, 0.5 mAh cm^−2^.

The polarization contributions of distinct electrode processes during zinc alloying processes were revealed by distribution of relaxation times (DRT) analysis of electrochemical impedance spectroscopy (EIS). Ex situ EIS data from a symmetric cell containing a protected anode clearly shows a gradual reduction in impedance with increasing cycling time within the 1^st^ cycle (Figure 4a). The corresponding DRT plots are shown in Figure 4b, in which the peaks at a time constant around 10^−4^ s can be assigned to ion transport across an anodic SEI or at the interfaces of alloys,[^45^, ^46^] those at 10^−3^ to 10^−1^ s derive from charge transfer kinetics at electrodes, while those from 10^0^ to 10^1.4^ s represent solid state diffusion process.[^47^, ^48^] The continuous reduction in both charge transfer kinetics and diffusion resistance indicate an enhancement in the mobility of zinc ions within the metallic interfacial layer as the alloying process proceeds. An important observation from the DRT plots is the emergence of a subtle peak at approximately 10^−4^ s, suggesting the gradual formation of the metallic alloy interphase on the surface of the Zn@Ag−In electrode. After 10 cycles a more pronounced peak appears at the same position, which means that a stable alloy interphase has been formed within the alloy layer (Figure S30 and Figure 4c). This observation is in alignment with the ex situ XRD results in Figure 2e, i.e., the alloying process has completed after around 10 cycles. In contrast, the charge transfer resistance for a pristine Zn||Zn symmetric battery is significantly larger than that of the modified one and no alloy interphase peak was found (Figure S31).

### Electrochemical Performance of Zn Metal Cells

Finally, long‐term cycling performance of symmetric, half and full cells containing the Zn@Ag−In electrode were tested. Figure 4d and Figures S32–35 show the CEs of Cu||Zn half cells. While the cells containing a modified anode initially displayed a lower CE, likely due to the ongoing alloying process (Ag+Zn→Ag_x_Zn_y_), after the metallic alloy interphase formation had stabilized the electrodes demonstrated an extremely high CE value, reaching up to 99.8 %, and maintained stable potential beyond 1400 cycles (Figure 4e).

Symmetric cells, as illustrated in Figure 4f, showed that an unprotected electrode cycled well at a lower potential of around 60 mV in the initial stage at 1 mA cm^−2^, 0.5 mA cm^−2^, however, after 800 cycles it encountered a short circuit. In contrast, the cell using the protected electrodes with various sputtering durations all showcased a markedly extended lifespan, in which the sample of 200 s offered the longest cycle life of over 8000 cycles. The cycling stability of our designed electrodes outperforms previous reports of single‐heterometallic protection strategies (Figure S36 and Table S4). Although a higher initial overpotential was found in initial cycles, it gradually reduced to 32 mV (Figure S37) and maintained stability throughout the cycles. The observed decrease in polarization can be attributed to the gradual alloying process, which enhances ionic mobility and reduces charge transfer resistance, aligning with findings from the DRT analysis.

**Figure 5:**
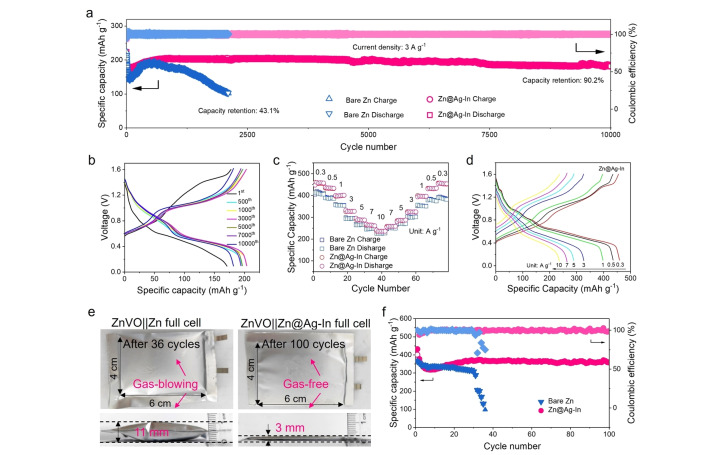
Electrochemical performance of full cells. a) Long‐term cycling performance of full ZVO||Zn coin cells with the two electrodes at a current density of 3 A g^−1^. b) The corresponding charge/discharge curves of the full cell with Zn@Ag−In electrode at various cycles. c) Rate performance of the full batteries with both electrodes. d) The corresponding charge/discharge curves of the full cell with Zn@Ag−In electrode at various current rates. e) The digital photographs of top views and cross sections of the cycled pouch cells. f) Long‐term cycling performance of full pouch cells with the two electrodes at a current density of 0.5 A g^−1^.

The viability of the Zn@Ag−In electrode was further verified in zinc vanadate (ZVO)||Zn full cells. As shown in Figure 5a, the full cells with a pristine zinc foil suffered a rapid capacity degradation to only 43.1 % after around 2000 cycles. However, when tested with an alloy anode, the full coin cells showed an ultra‐long lifespan of over 10,000 cycles with a superior capacity retention of 90.2 %, even at a high current density of 3 A g^−1^. The corresponding charge/discharge curves in Figure 5b further demonstrate the cycle stability. The lifespan of the full cell marks the highest durability achieved to date in comparison with prior research, including zinc metal protection and electrolyte modification (Table S5). The full battery with an alloy anode also exhibits better cycle reversibility at various current densities, due to less by‐product accumulation (Figures 5c, 5d and Figure S38). Finally, to advance the practical application of the novel ZMA, we assembled a full pouch cell (Figure 5e), which also showed good stability and a high‐capacity retention of 80.6 % after 100 cycles (Figure 5f). Importantly, while the initial thickness of the pouch cells was equivalent, the post‐cycling thickness of the cycled pouch cell with the protected anode measured approximately 3 mm, in sharp contrast to the 11 mm thickness observed with a bare anode, attributable to H_2_ generation during cycling. This means that the composite ZMA′s capacity to suppress side reactions is effective in a full cell configuration, showing strong agreement with the HER results in Figure 3h. The cycling stability of both symmetric and full cells with our designed electrode therefore highlights the efficacy of our design strategy based initially on theoretical calculations.

## Conclusion

In summary, we have engineered an in situ evolved metallic alloy interlayer for aqueous Zn battery anodes that is both scalable, as it is deposited via a simple magnetron sputtering process, and highly effective at negating the formation of Zn dendrites and halting the progression of detrimental side reactions. While initially composed of metallic Ag and In, this dual‐heterometallic protective layer spontaneously transforms during early electrochemical cycling to contain both AgZn_3_ to control Zn deposition morphology and In to promote Zn‐ion transport within this alloy interphase, greatly improving the reversibility of metallic zinc anode and avoiding interfacial deposition of metallic Zn. Batteries containing this protected Zn anode exhibited markedly enhanced performance: symmetric cells achieved over 8000 cycles with a high CE of 99.8 % and full cells surpassed 10,000 cycles with a capacity retention of 90.2 %. This methodology for metallic interface design therefore offers a route to further advance the commercial viability of highly sustainable and safe AZIBs, as well as other multivalent metal batteries.

## Conflict of Interests

The authors declare no conflict of interest.

1

## Supporting information

As a service to our authors and readers, this journal provides supporting information supplied by the authors. Such materials are peer reviewed and may be re‐organized for online delivery, but are not copy‐edited or typeset. Technical support issues arising from supporting information (other than missing files) should be addressed to the authors.

Supporting Information

## Data Availability

The data that support the findings of this study are available from the corresponding author upon reasonable request.
