# Supplementary material for: Operando Evolution of a Hybrid Metallic Alloy Interphase for Reversible Aqueous Zinc Batteries
Source: Angew Chem Int Ed Engl. 2024 Dec 23;64(5):e202416047. doi: 10.1002/anie.202416047 (PMC11773305; doi:10.1002/anie.202416047)
Supplement: Supplementary file 1 — Supporting Information [file ANIE-64-e202416047-s001.pdf]

Angewandte Chemie
Eine Zeitschrift der Gesellschaft Deutscher Chemiker

Supporting Information

Operando Evolution of a Hybrid Metallic Alloy Interphase for Reversible Aqueous Zinc Batteries

M. Liu, K. Yang, Q. Xie, N. Hu, M. Zhang, R. Chen, W. Zhang, J. Zhang, F. Shao, H. He, R. Soni, X. Guo, J. Yang, G. He, F. Pan, L. Yao*, T. S. Miller**

Supplementary Information

Operando Evolution of a Hybrid Metallic Alloy Interphase for Reversible Aqueous Zinc Batteries

Mingqiang Liu, Kai Yang, Qiming Xie, Nantao Hu, Mingzheng Zhang, Ruwei Chen, Wei Zhang, Jichao Zhang, Feng Shao, Hongzhen He, Roby Soni, Xiaoxia Guo, Jinlong Yang, Guanjie He, Feng Pan, Lu Yao*, Thomas S. Miller**

[*] Dr. L. Yao, Dr. M. Liu
School of Materials Science and Engineering, Shanghai Institute of Technology, Shanghai 201418 (P. R. China)
E-mail: luyao@sit.edu.cn
Prof. T.S. Miller, Dr. M. Liu, H. He, Dr. R. Soni, Dr. X. Guo
Electrochemical Innovation Lab, Department of Chemical Engineering, University College London, London, WC1E 7JE, UK
E-mail: t.miller@ucl.ac.uk
Prof. F. Pan, Dr. L. Yao, Dr. M. Liu, Q. Xie, M. Zhang,
School of Advanced Materials, Peking University Shenzhen Graduate School, Shenzhen 518055 (P. R. China)
E-mail: panfeng@pkusz.edu.cn
Dr. K. Yang
Advanced Technology Institute, Department of Electrical and Electronic Engineering, University of Surrey, Guildford, Surrey, GU2 7XH, UK
Prof. N. Hu
Key Laboratory of Thin Film and Microfabrication Technology (Ministry of Education), School of Electronics, Information and Electrical Engineering, Shanghai Jiao Tong University, Shanghai 200240 (P. R. China)
Dr. R. Chen, Dr. W. Zhang, Dr. J. Zhang, Prof. G. He
Department of Chemistry, University College London, London, WC1E 7JE, UK
Dr. F. Shao
School of chemistry and chemical engineering, Shanghai Jiao Tong University, Shanghai 200240 (P. R. China)
Prof. J. Yang
Guangdong Research Center for Interfacial Engineering of Functional Materials, College of Materials Science and Engineering, Shenzhen University, Shenzhen 518060 (P. R. China)

Experimental section:

1.1 Synthesis of $Zn_{0.27}V_2O_5 \cdot nH_2O$ cathode material

The $Zn_{0.27}V_2O_5 \cdot nH_2O$ nanobelts were synthesized according to a previously reported method. At first, 3 mM V_2O_5 and 2.33 mM zinc acetate were mixed in a solution containing 70 ml of water/acetone (with a volume ratio of 14:1) and 0.3 ml of nitric acid. The mixture was thoroughly dispersed, and then transferred into a 100 ml Teflon-lined stainless-steel autoclave and heated at 180 ºC for 24 hours. Subsequently, the synthesized product was filtered and washed three times with deionized water and ethanol. Finally, the product was dried in an 80 ºC vacuum oven overnight.

1.2 Preparation of cathode

Mixing and stirring vigorously of the above synthesized $Zn_{0.27}V_2O_5 \cdot nH_2O$ nanobelts with polyvinylidene fluoride (PVDF) and acetylene black at a mass ratio of 7:2:1 in N-methy pyrrolidone (NMP) solvent. The prepared slurry was then evenly coated on Titanium (Ti) foil by a blade and dried in a vacuum oven at 80 °C overnight. The loading of the cathode material was approximately 1.0 mg cm^{-2}.

1.3 Preparation of anode

The Zn foil was immersed in acetone solvent and cleaned with ultrasound. Then a magnetron sputtering instrument utilizing direct current was used to deposit silver and indium (using an Ag-In target with a ratio of 3:1) on the surface of Zn foil for different durations (15 s, 30 s, 100 s, 200 s, 300 s). The sputtering parameters included a power of 150 W, a working pressure of approximately 1 Pa, an Argon gas flow of 20-30 sccm, and a target-to-substrate distance of 75 mm. After deposition, the sputtered Zn foil was cut into discs with a diameter of 12 mm for coin cells and 3×3 cm square for pouch cells.

1.4 Battery assembly and electrochemical measurements

Coin cells (CR2032) were assembled using the above cut Zn plates and $Zn_{0.27}V_2O_5 \cdot nH_2O$ cathodes with a diameter of 12 mm. A flexible pouch battery was packaged with Zn anode and $Zn_{0.27}V_2O_5 \cdot nH_2O$ cathode (3×3) with the same size. The assembled Zn||Zn symmetric cells and Zn||Cu half cells were used to measure the stripping/plating behaviors and CE of zinc at various current densities, separately. For the HER measurement and Tafel plot was collected in a three-electrode system with Zn foil as the working electrode, a Pt sheet as counter electrode and an Ag/AgCl reference electrode. The above tests were all carried out on a CHI604E electrochemical workstation (Chenhua, Shanghai).

1.5 Characterization

X-ray diffraction (XRD,) patterns were collected on a D8 ADVANCE (Bruker Co., Germany) using Cu Kα, λ=1.541 Å, with 2θ from 5° to 90°. The surface morphology and elements distribution of the samples were observed under a Scanning electron microscope (SEM, SUPRA 55 SAPPHIRE, Carl Zeiss AG, Germany) and energy dispersive spectrometer (EDS,51-XMX1004, Oxford Instruments Inc., America), separately. Transmission electron microscopy (TEM) was used to observe the morphology of SEI of cycled samples. The compositional information of samples was characterized by X-ray photoelectron spectroscopy (XPS, Thermo Fisher ESCALAB 250Xi).

Theoretical calculations:

Lattice mismatch ratio (R) was calculated based on the formula of ($R = |\frac{d_{m(002)} - d_{Zn(002)}}{d_{Zn(002)}}|$)[1], in which d-space is the distance of the two adjacent atoms on (002) crystal planes of the metals and alloys reported in zinc metal protection.

First-principles calculations were systematically conducted through the application of Density Functional Theory (DFT) utilizing the Vienna Ab Initio Simulation Package (VASP) code, specifically version 5.3.5 [2]. Electron-ion interactions were delineated employing projector augmented wave (PAW) pseudopotentials, and the Perdew–Burke–Ernzerhof (PBE) generalized gradient approximation functional, as documented in the VASP database (potpaw_PBE.54) [3]. The consideration of Van der Waals interaction was implemented at the DFT-D3 level with a zero-damping function [4]. The Brillouin zone was meticulously sampled utilizing a Gamma-centered K-point mesh, established through VASPKIT, with a Kmesh-Resolved Value of 0.04×2πÅ^(-1) [5]. An energy cutoff of 500 eV was judiciously employed for the absorption calculations. To ensure computational accuracy, convergence criteria were established for optimization processes, necessitating a threshold of 10^(-5) eV for electronic self-consistency iterations and 0.02 eV for ionic relaxation loops. Furthermore, a vacuum layer of 15 Å was incorporated along the z-direction to preempt periodic interactions and enhance the accuracy of the calculations.

Zinc surfaces were explored with a focus on the Zn (002), (100), and (101) facets, which were modeled utilizing (4×4×1), (2×4×1), and (2×4×1) supercells, respectively, each with thicknesses varying between 9 and 12 Å. Similarly, the $AgZn_3$ structures were subject to exploration on analogous low-index surfaces (002), (100), and (101), employing (2×2×1) supercells, while maintaining dimensions and thicknesses in congruence with the Zn surfaces.

To extend the investigation, $AgZn_3$-In surfaces were generated by strategically placing an In4 cluster atop the previously mentioned $AgZn_3$ slabs. This systematic approach establishes a uniform framework for comparing the adsorption characteristics of individual Zn atoms on Zn, $AgZn_3$, and $AgZn_3$-In surfaces. This methodical exploration allows for a comprehensive assessment of the adsorption properties across different surface configurations, facilitating a nuanced understanding of their respective behaviors.

Zn adsorption was investigated across various cases by adsorbing a single Zn onto these surfaces, Subsequent to adsorption, comprehensive geometry optimization and electronic calculations were conducted, adhering rigorously to the previously stipulated convergence criteria. The stability of adsorption was evaluated through the determination of binding energy (BE), formulated as BE = E(Zn−S)−E(Zn−atom)−E(S), where E(Zn−S), E(Zn−atom), and E(S) denote the total energies of the Zn atom adsorbed on the slab surface, the isolated gas-phase Zn atom, and the pristine slab surface, respectively. A more negative BE value indicates heightened adsorption strength, serving as a crucial criterion for assessing stability.

The charge differential density, pivotal for understanding electronic interactions, was computed using VASPKIT and subsequently visualized through the utilization of the Vesta software. Additionally, the energy barriers associated with Zn atom migration were quantified employing the Climbing Image Nudged Elastic Band (CI-NEB) method [6][7], providing insights into the dynamics and kinetics of Zn atom movement within the studied systems. This multifaceted analytical approach contributes to a comprehensive understanding of Zn adsorption behavior, encompassing both energetic and electronic considerations.

Discussion of XPS spectra:

According to the XPS spectra (Figure 2h, Figure S27 and Table S1-3) of a cycled electrode, the binding energies of the Zn-Ag alloy shift. From the pristine material Zn 2p spectrum we can see that the characteristic Zn $2p_{3/2}$ is at ~ 1021.7 eV, but the alloy Zn-Ag peak is shifted to a slightly lower binding energy of approximately 1020.8 eV. While in Ag 3d spectrum, the Ag $3d_{5/2}$ peak is initially located at ~368.0 eV, but its binding energy within the alloy Ag-Zn shifted to an average value of 368.9 eV.

Characterization of the sample:

We modified bare zinc foils by direct current sputtering of silver and indium. The surface was rough at low sputtering times but was found to get smoother as deposition time increased (Figure S11). Energy dispersive spectroscopy (EDS) images confirmed the uniformity of the

elemental distribution of Ag and In. For electrochemical tests and onward characterization, Zn sputtered with Ag and In for 200 s (corresponding to ~0.3 μm, Figure S12) was selected as the electrode as this was found to offer the longest life span within a symmetric cell (Figure S13). From the X-ray diffraction (XRD) patterns of the uncycled electrodes in Figure S14, a distinct peak at 38.0^{o} can be seen, which can be assigned to crystalline Ag. However, no distinct peaks can be ascribed to In. X-ray photoelectron spectroscopy (XPS) characterization (Figure S15) on the surface of Zn@Ag-In electrode, showed distinct peaks at 445 and 452.5 eV attributed to In and In_2O_3, implying that part of the In on the surface has been oxidized. XPS analysis of sputtered Ag and In on SiO_2 under the same conditions (SiO_2@Ag-In) showed a similar result to those above (Figure S16). This suggests that in the as-deposited interface layer the In exists in an amorphous state within the composite metallic interlayer on the Zn metal surface. The atomic ratio of Ag to In is close to 3:1, based on XPS analysis (Figure S17).

Supplementary figures

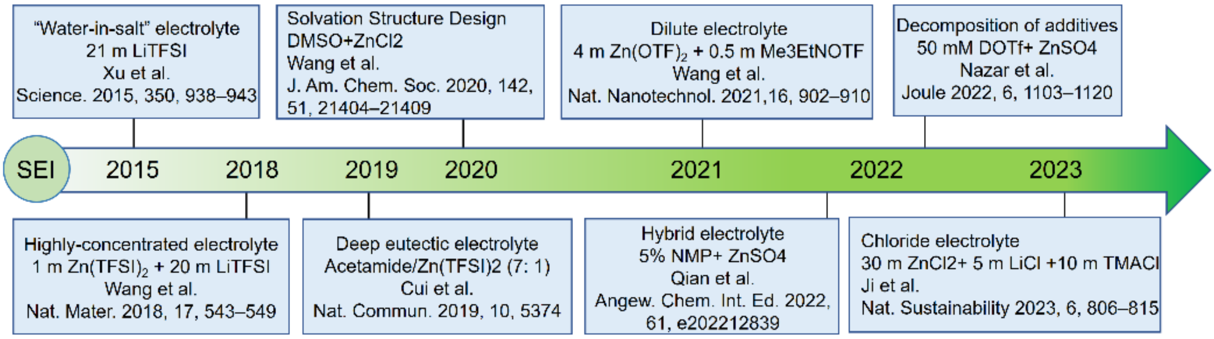

Figure S1. Brief history of in situ SEI design methods for aqueous batteries[8–15].

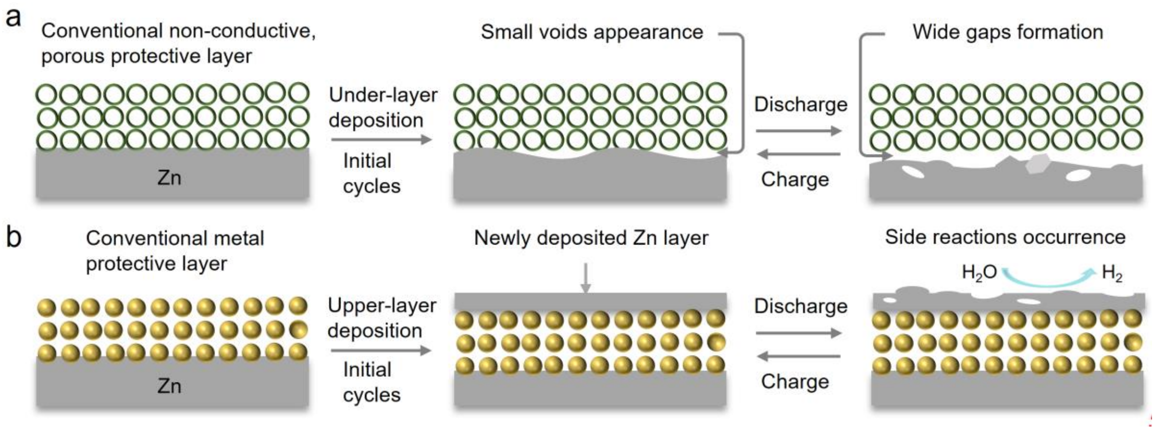

Figure S2. Schematic diagram of the evolution of **a,** conventional non-conductive, porous protective layer, **b**, conventional metallic protective layer during zinc stripping/plating processes.

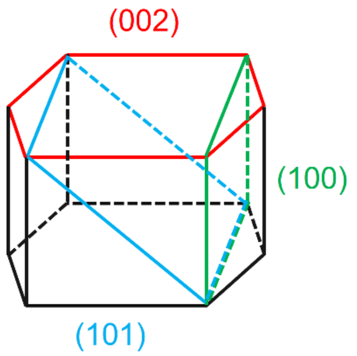

Figure S3. Schematic diagram of the crystal planes of Hexagonal Zn.

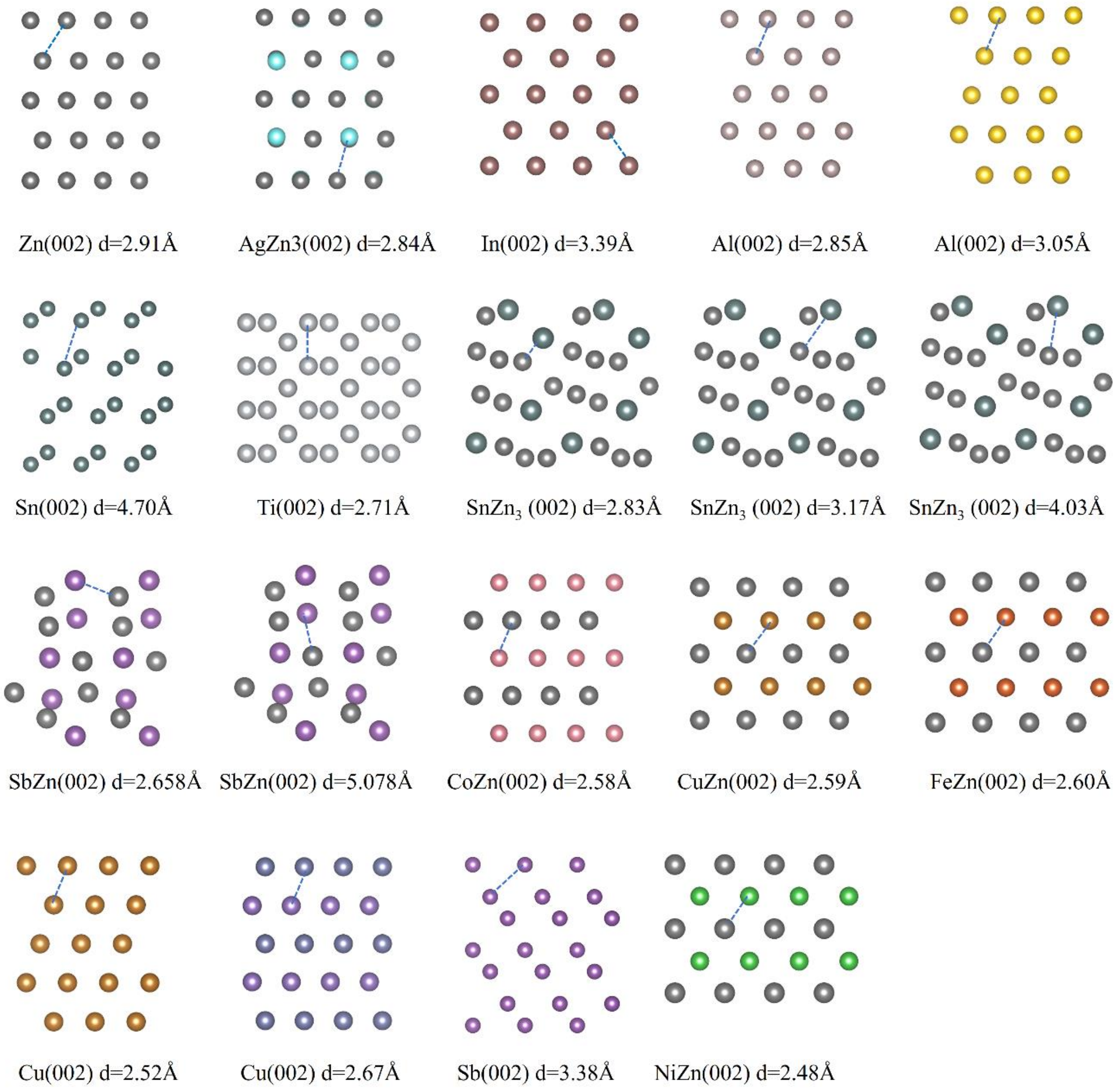

Figure S4. Measurement of the d-space of two adjacent atoms on (002) crystal planes of the metals and alloys reported in zinc metal protection.

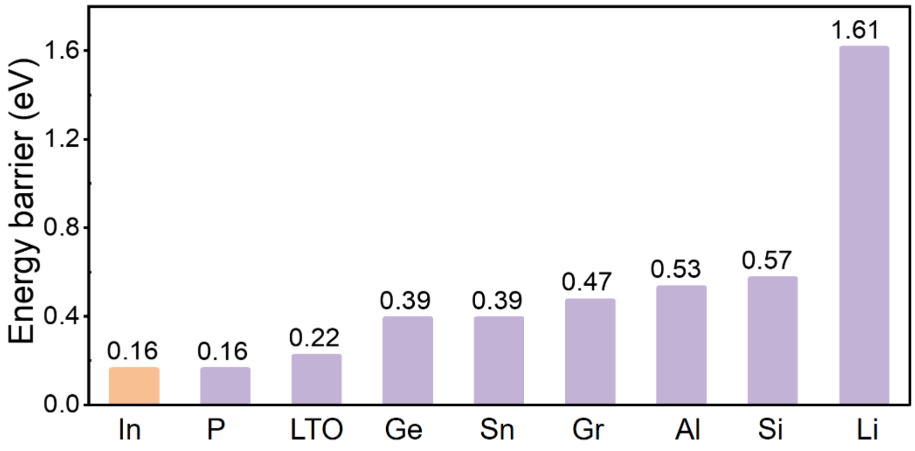

Figure S5. Migration energy barriers for Li transport in different solid-state materials, including In, P, LTO, Ge, Sn, Gr, Al, Si, Li [16–22].

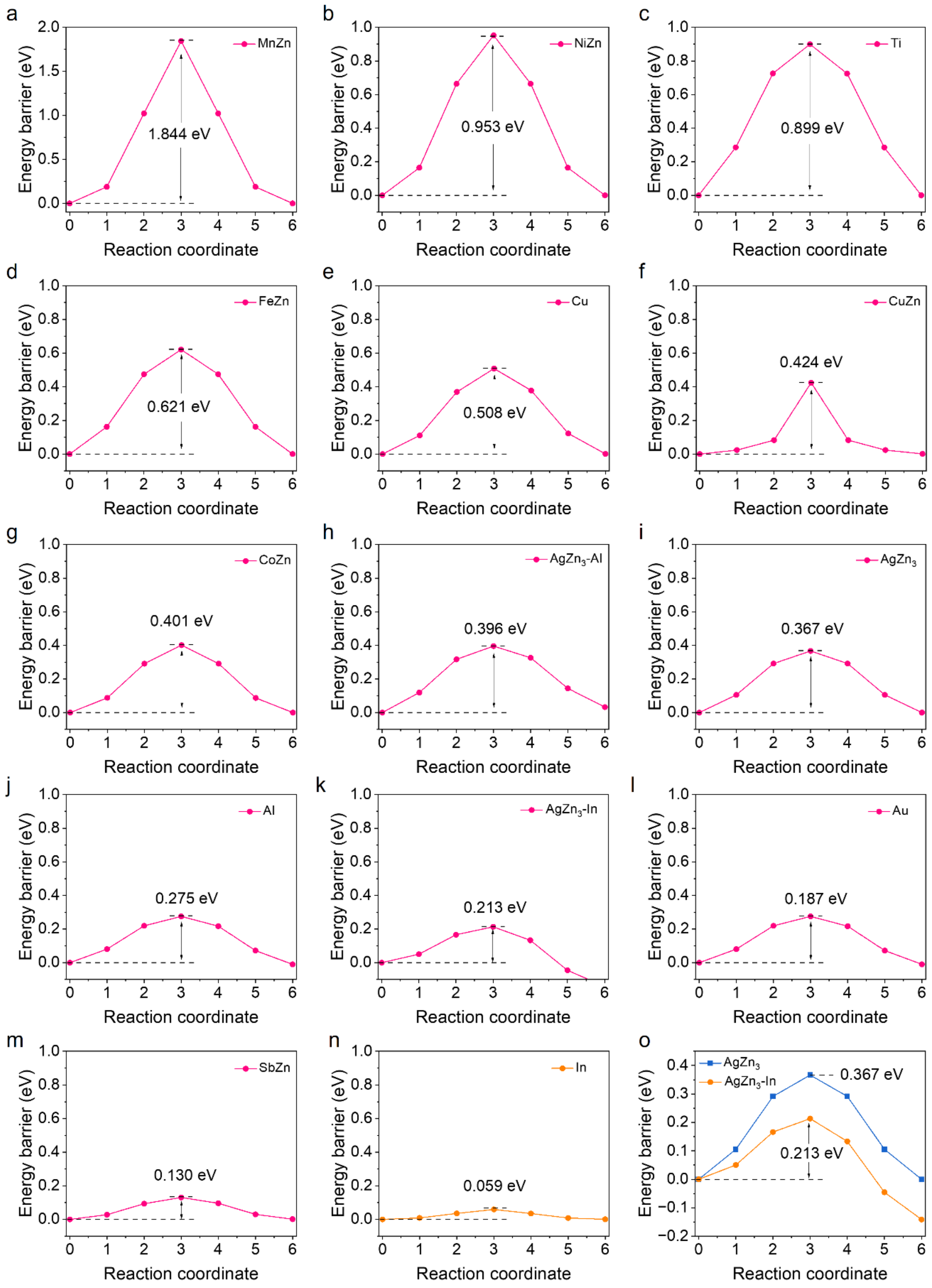

Figure S6. Migration energy barriers for zinc transport in the lattice vacancies of different metals and alloys.

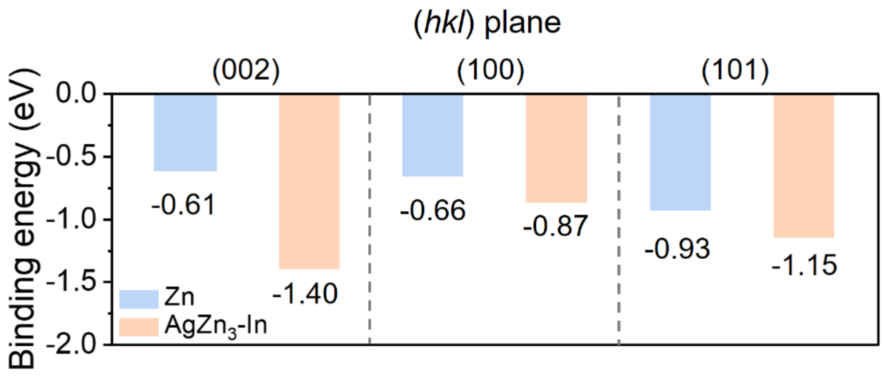

Figure S7. Binding energy of zinc atom on (002), (100) and (101) crystal planes of Zn and $AgZn_3$-In.

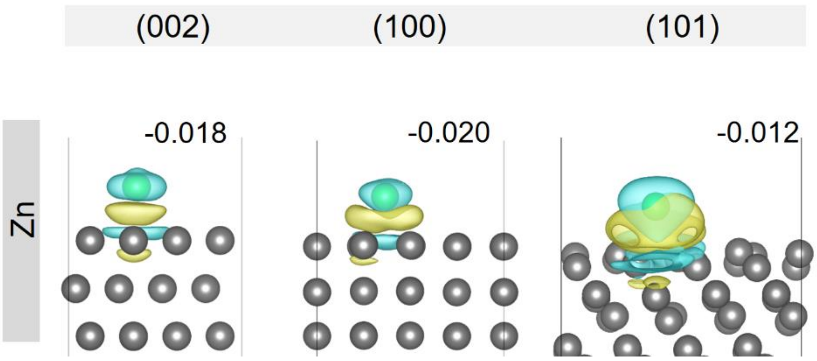

Figure S8. Differential charge density of Zn atom on (002), (100) and (101) crystal planes of Zn.

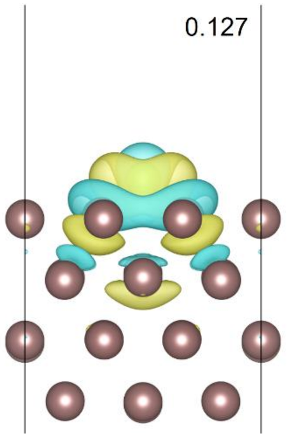

Figure S9. Differential charge density of Zn atom on (002) crystal plane of In.

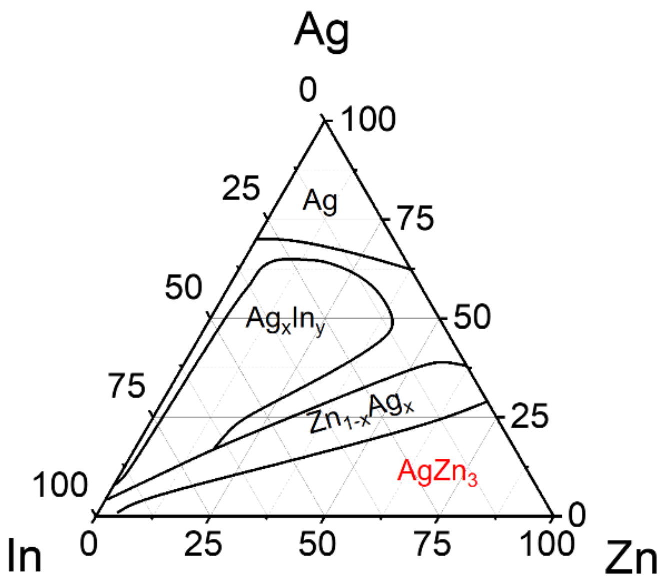

Figure S10. Ternary phase diagram of Zn-Ag-In [23].

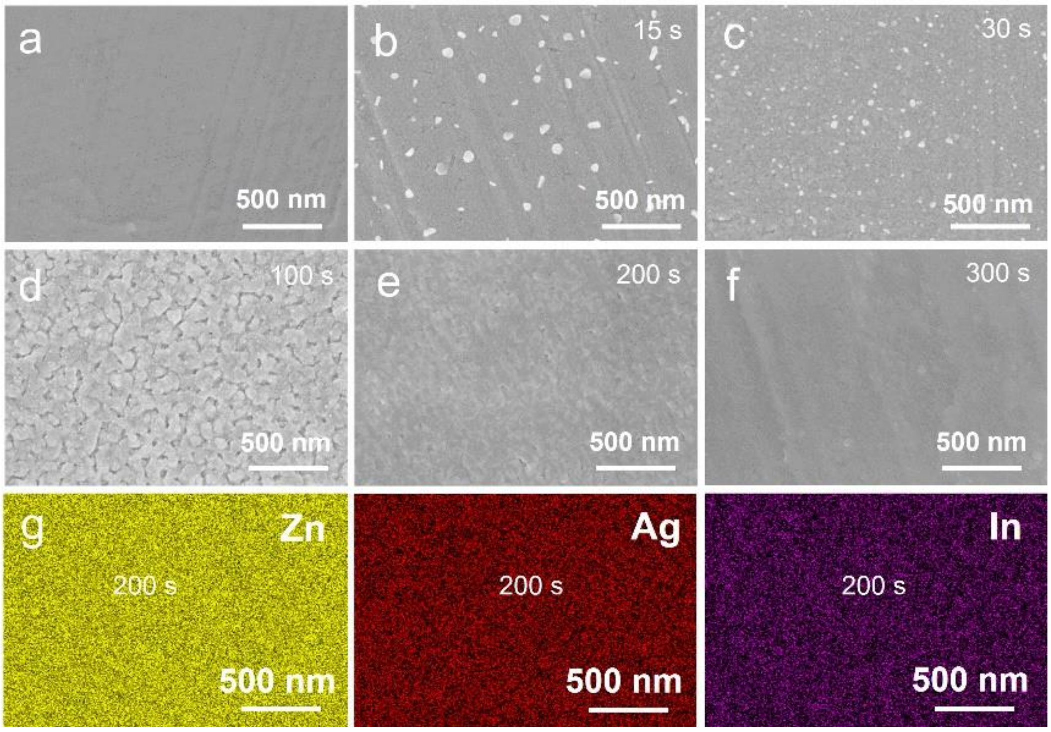

Figure S11. SEM images of the surface of (a) bare zinc foil; Zn foil after sputtering Ag and In (b) 15 s, (c) 30 s, (d) 100 s, (e) 200 s, (f) 300 s, respectively; (g) EDS of the electrode Zn@Ag-In with the sputtering time of 200 s.

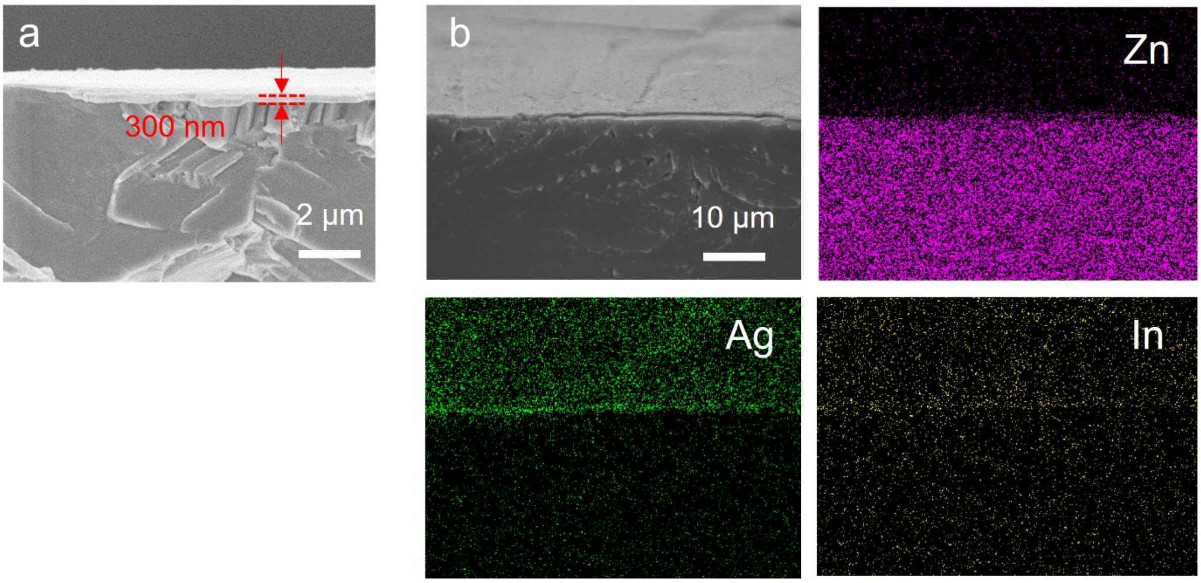

Figure S12. SEM images and EDS of the cross section of the Zn@Ag-In electrode with a sputtering time of 200 s.

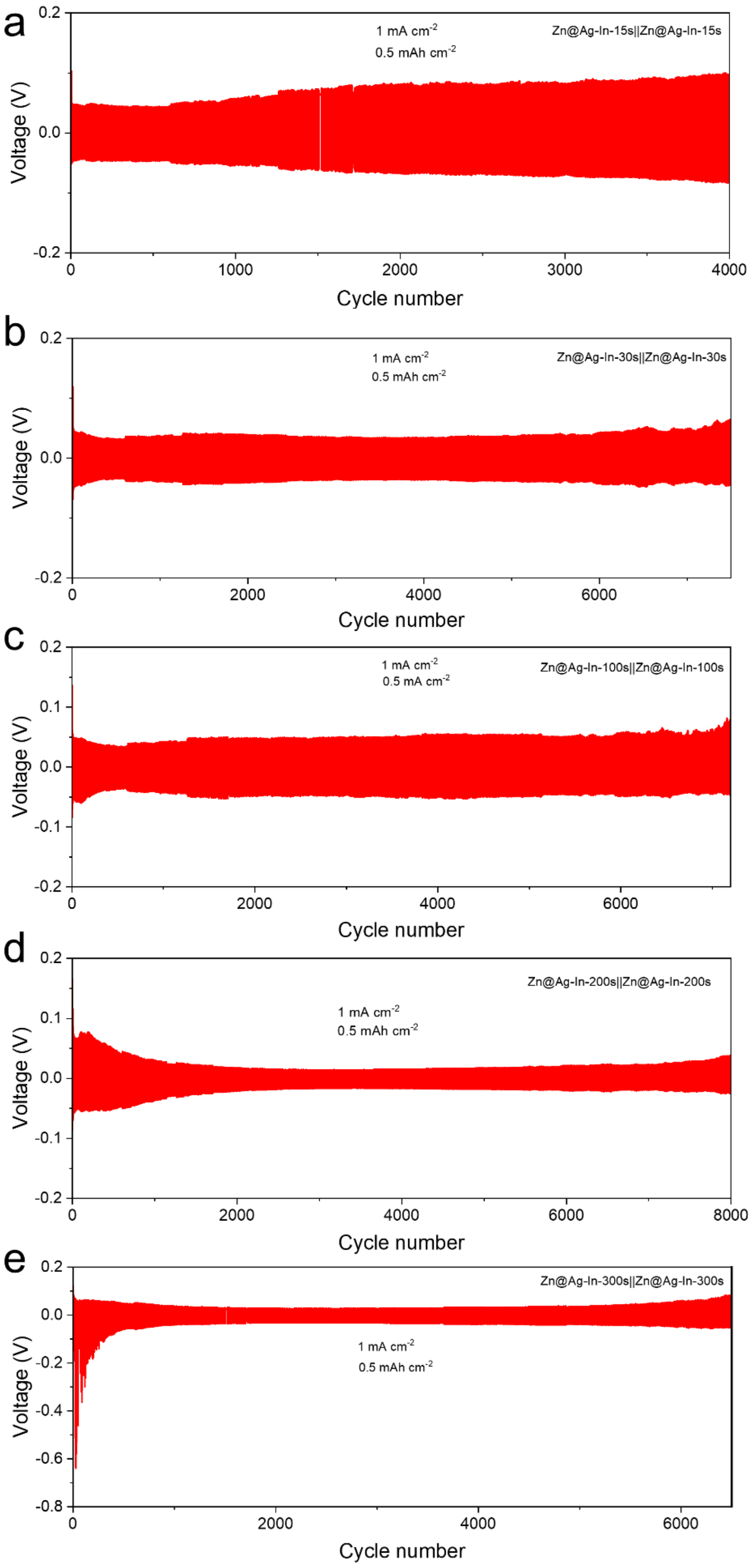

Figure S13. Long-term cycling performance of symmetric cells with Zn@Ag-In electrodes at various sputtering time of 15 s, 30 s, 100 s, 200 s, 300 s, respectively, at 1 mA cm^{-2}, 0.5 mAh cm^{-2}.

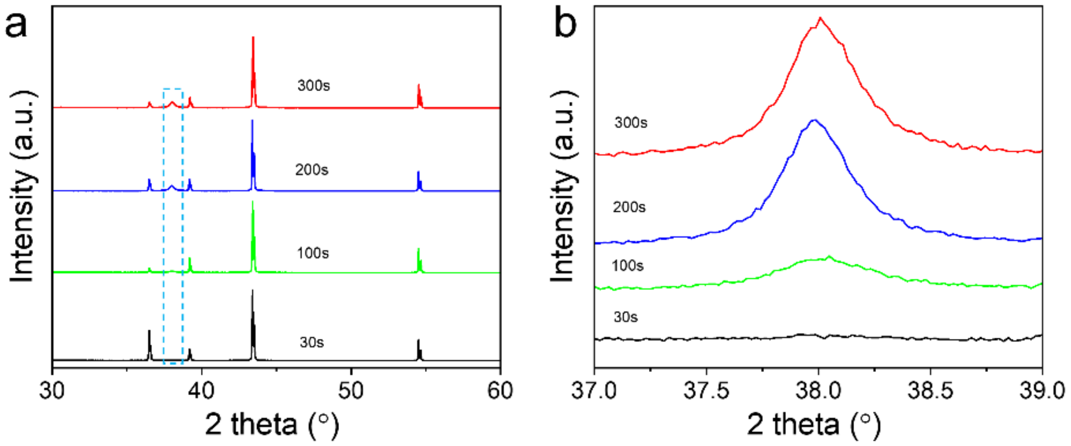

Figure S14. (a) XRD patterns of the sputtered electrodes, including 30 s, 100 s, 200 s, 300 s. (b) the magnified section of the XRD patterns from 37.0° - 39.0° in the dashed area of (a).

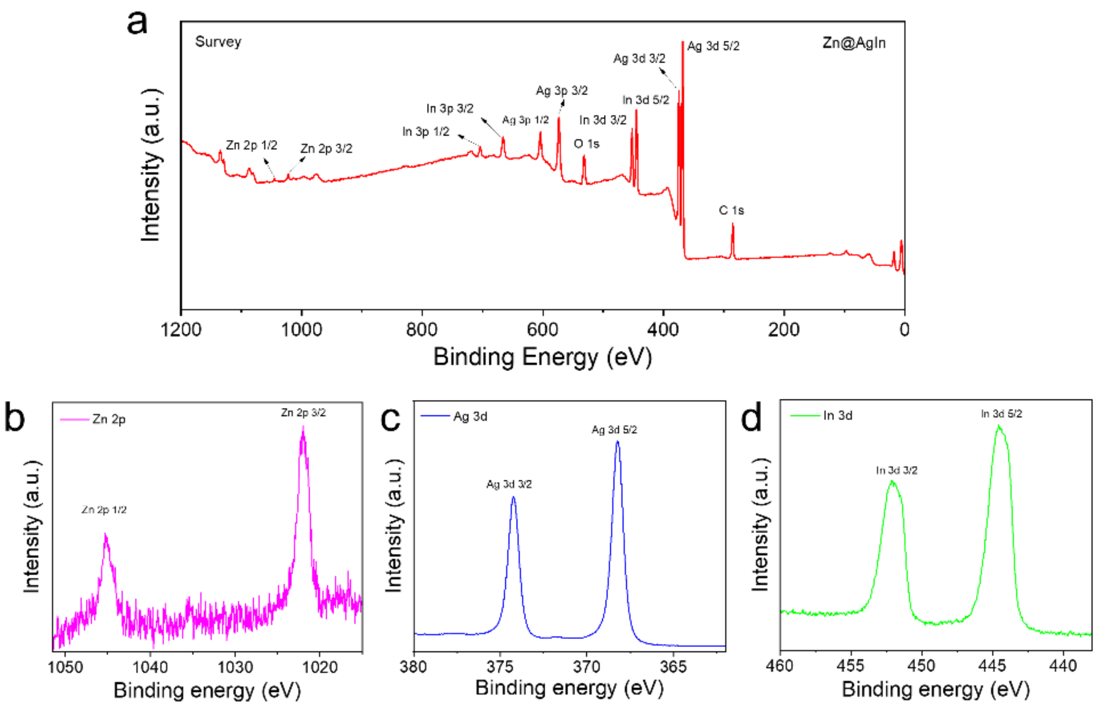

Figure S15. XPS spectra of (a) Zn@Ag-In, (b) Zn 2p, (c) Ag 3d and (d) In 3d.

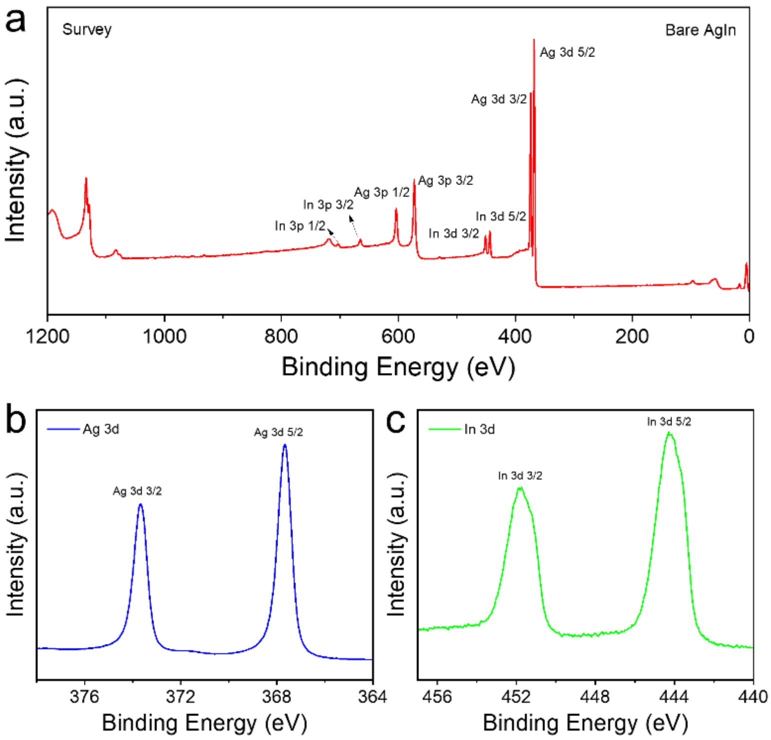

Figure S16. XPS spectra of (a) SiO_2@Ag-In, (b) Ag 3d and (c) In 3d.

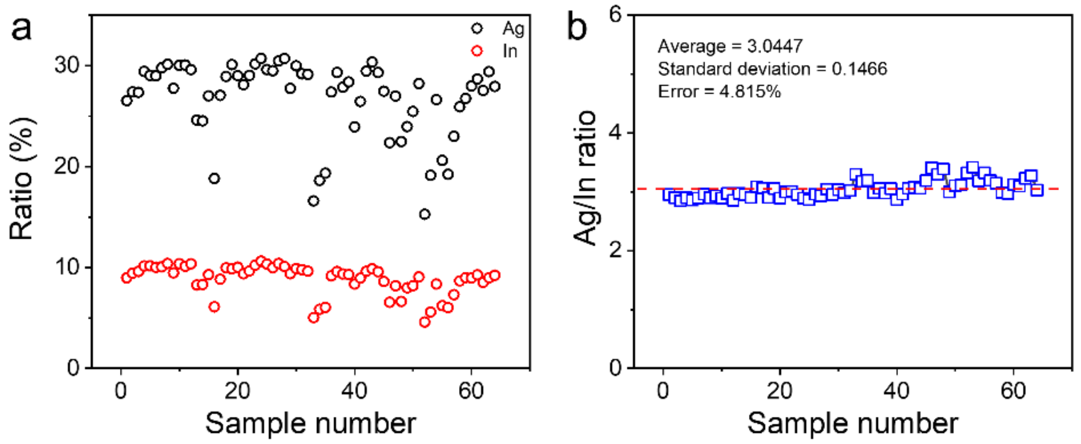

Figure S17. Atomic ratio of Ag and In on the surface of the Zn@Ag-In electrode by XPS analysis.

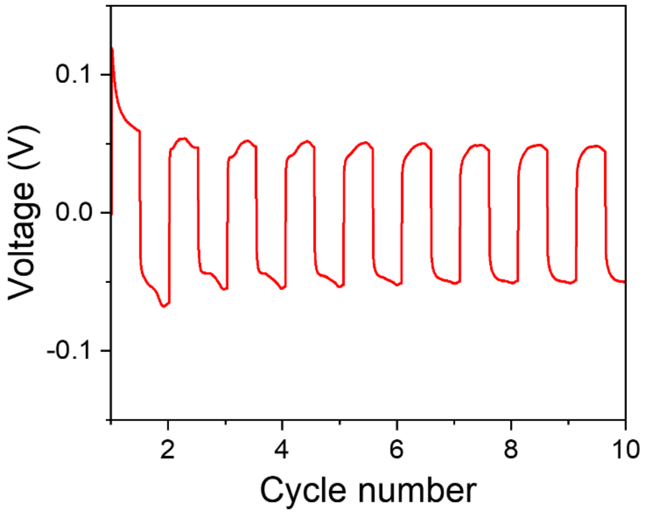

Figure S18. Cycling performance of symmetric cells with Zn@Ag-In electrodes at 1 mA cm^{-2}, 0.5 mAh cm^{-2}, followed by a ToF-SIMS test on the cycled electrode.

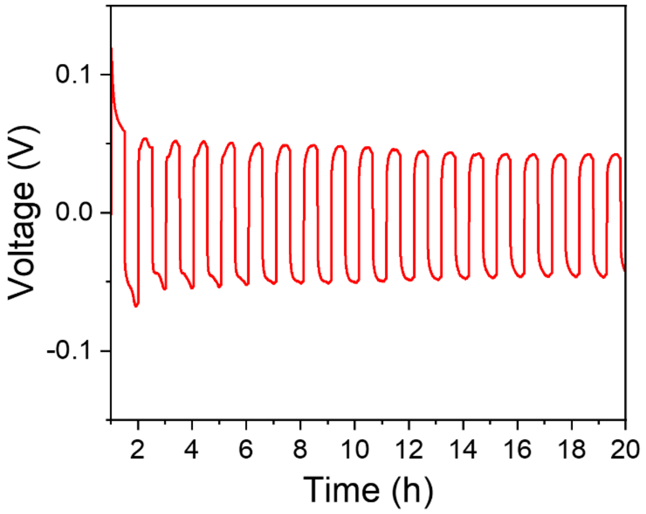

Figure S19. Cycling performance of symmetric cells with Zn@Ag-In electrodes at 1 mA cm^{-2}, 0.5 mAh cm^{-2}, followed by an observation under FIB-SEM on the cycled electrode.

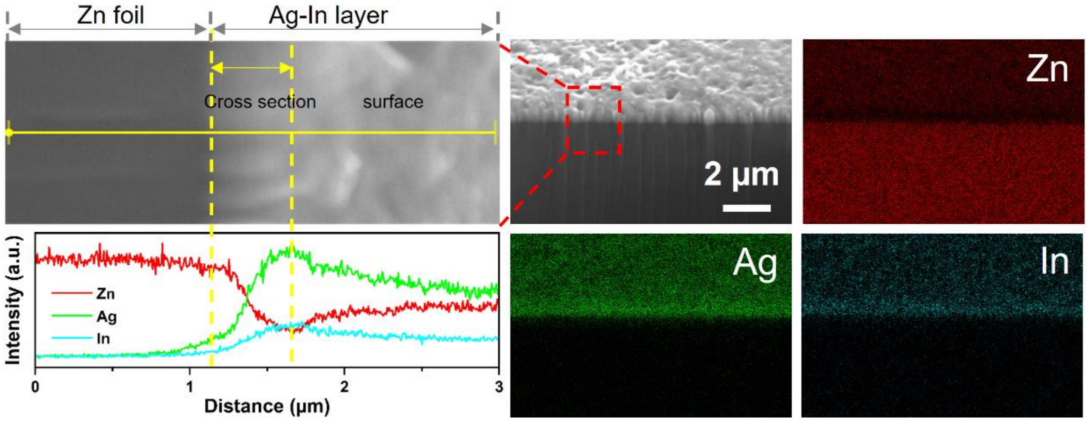

Figure S20. Fib-SEM images and EDS mapping of the cross section of the Zn@Ag-In electrode in a symmetric cell after 20 cycles. Intensity-distance curves show the elemental distribution of Zn, Ag and In from the bottom to the surface of the electrode.

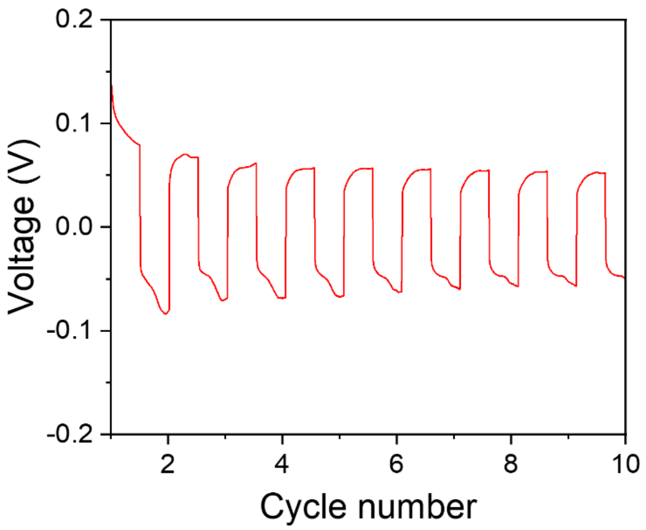

Figure S21. Cycling performance of symmetric cells with Zn@Ag-In electrodes at 1 mA cm^{-2}, 0.5 mAh cm^{-2}. The cycled electrode was then analyzed using a combination of the techniques including HRTEM, EDS, XRD and XPS.

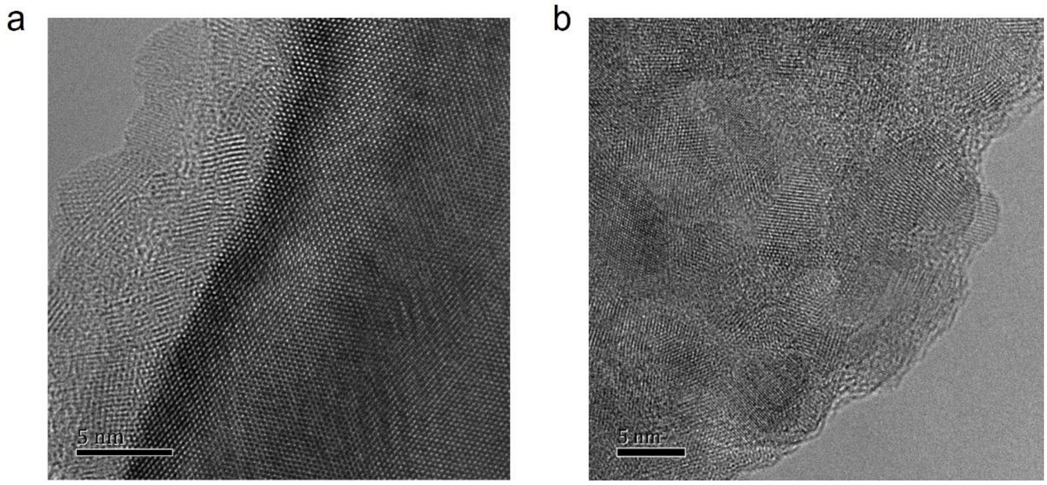

Figure S22. HRTEM images of (a) cross section and (b) top view of the cycled Zn@Ag-In electrode, original Figure 2c and 2e.

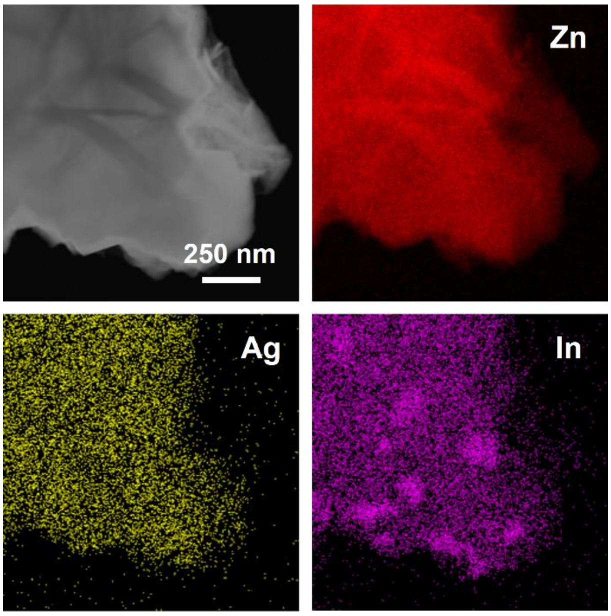

Figure S23. EDS mapping of the Zn@Ag-In electrode after 10 cycles.

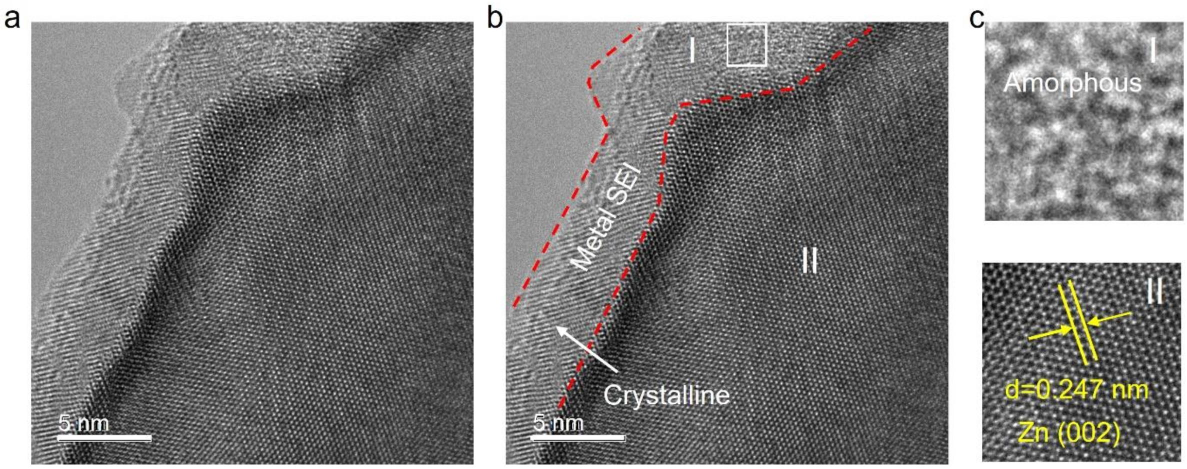

Figure S24. HRTEM images of cross section of the cycled Zn@Ag-In electrode.

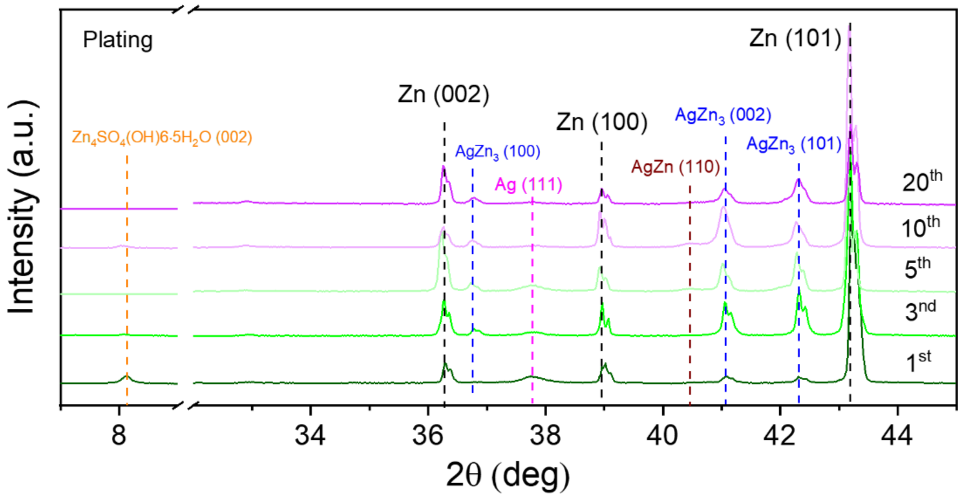

Figure S25. Ex situ XRD patterns of Zn@Ag-In electrodes in symmetric cells at different stages of cyclic plating.

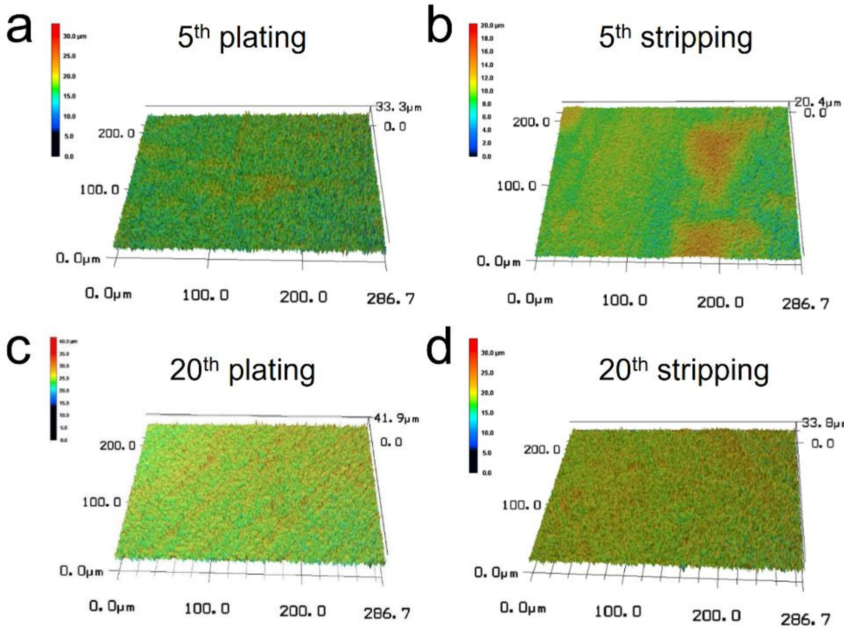

Figure S26. 3D CLSM images of Zn@Ag-In electrode after 5th (a, b) and 20th (c, d) plating/stripping.

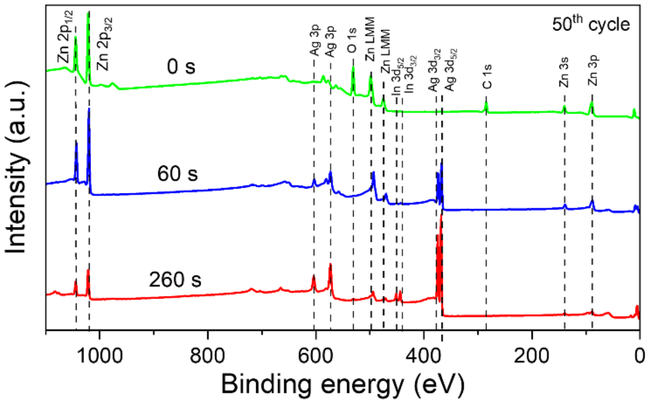

Figure S27. XPS spectra of Zn@Ag-In electrode after 50 cycles with the etching time of 0 s, 60 s and 260 s.

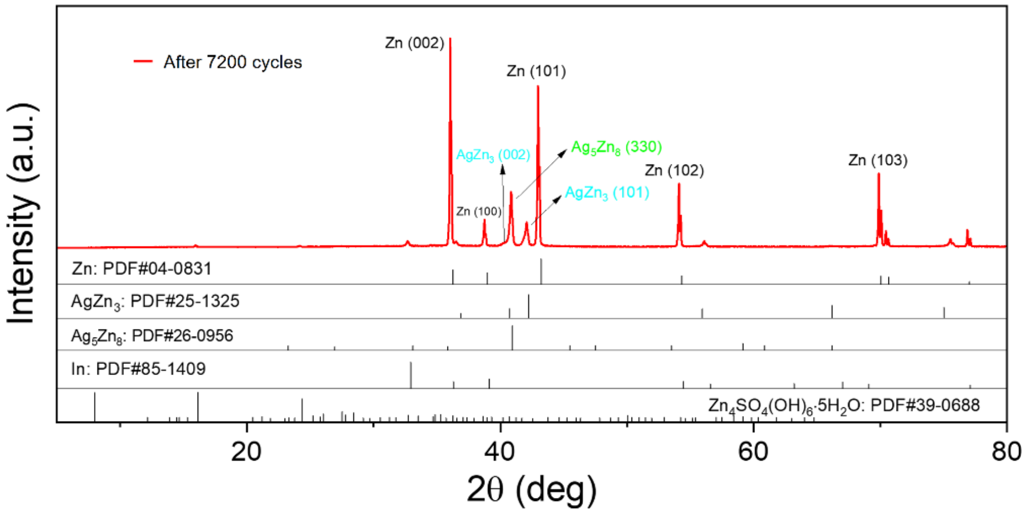

Figure S28. XRD patterns of the cycled Zn@Ag-In electrode in a symmetric battery after 7200 cycles and Zn, $AgZn_3$, Ag_5Zn_8, In and $Zn_4SO_4(OH)_6 \cdot 5H_2O$ standardized XRD patterns.

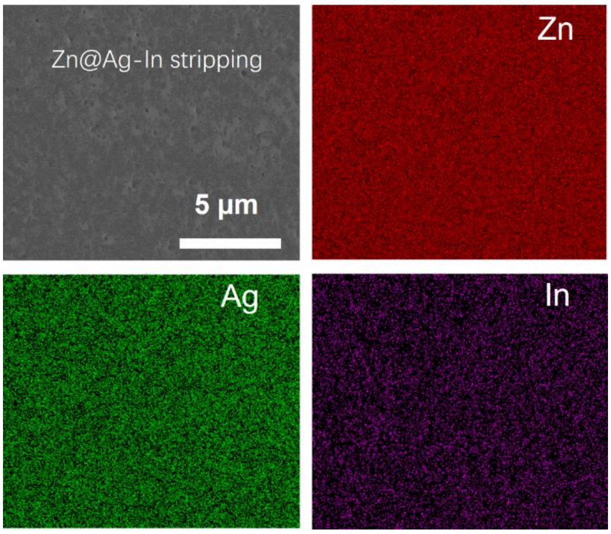

Figure S29. SEM image and EDS mapping of a Zn@Ag-In electrode after stripping 100 cycles at a current density of 1 mA cm-2, 0.5 mAh cm^{-2}.

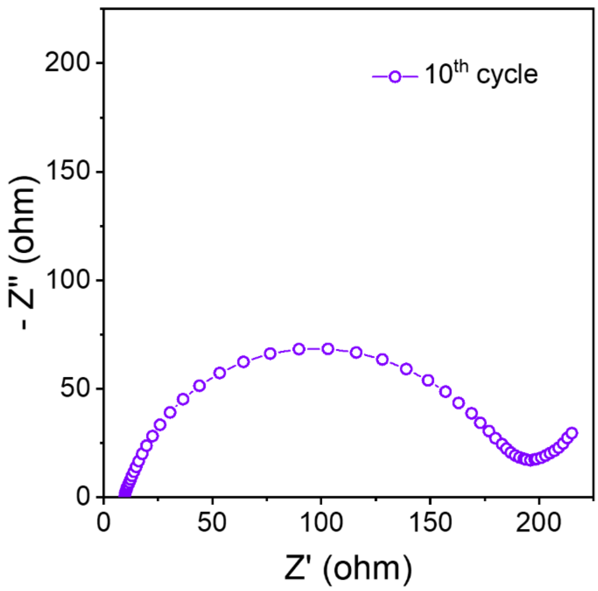

Figure S30. EIS plot of the Zn@Ag-In||Zn@Ag-In symmetric battery after 10 cycles.

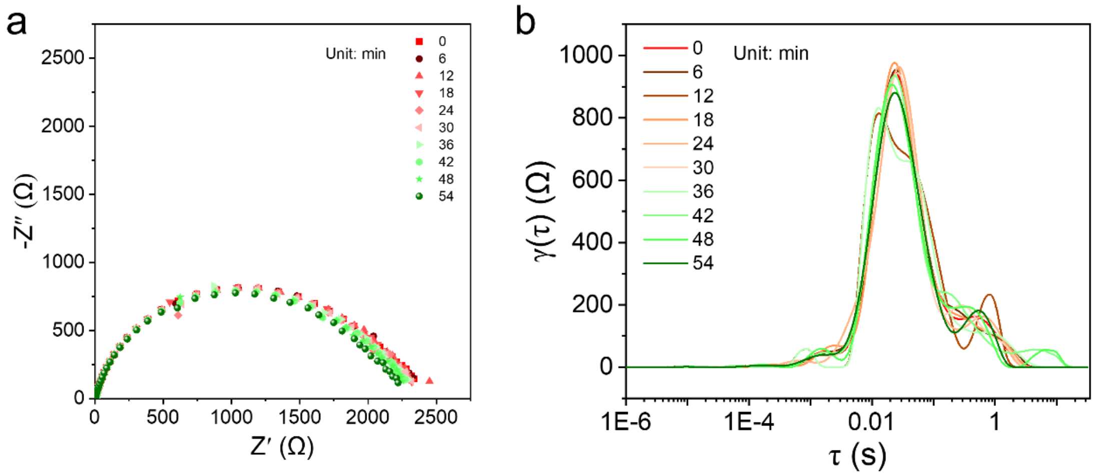

Figure S31. (a) EIS spectra and (b) the corresponding DRT plots of the Zn||Zn symmetric battery with different cycle times at 1st cycle (6 minutes per step).

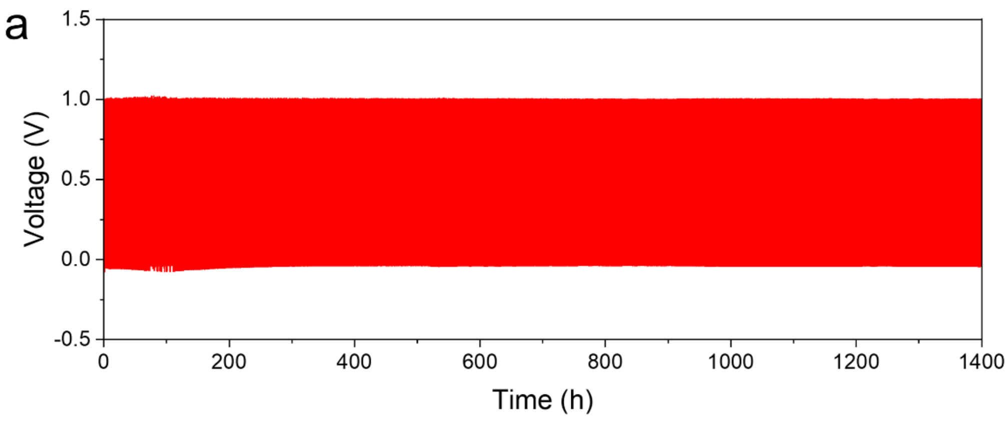

Figure S32. The voltage-time curves of Zn@Ag-In||Cu half cell with 3 M $Zn(OTf)_2$ at 1 mA cm^{-2} and 0.5 mAh cm^{-2}.

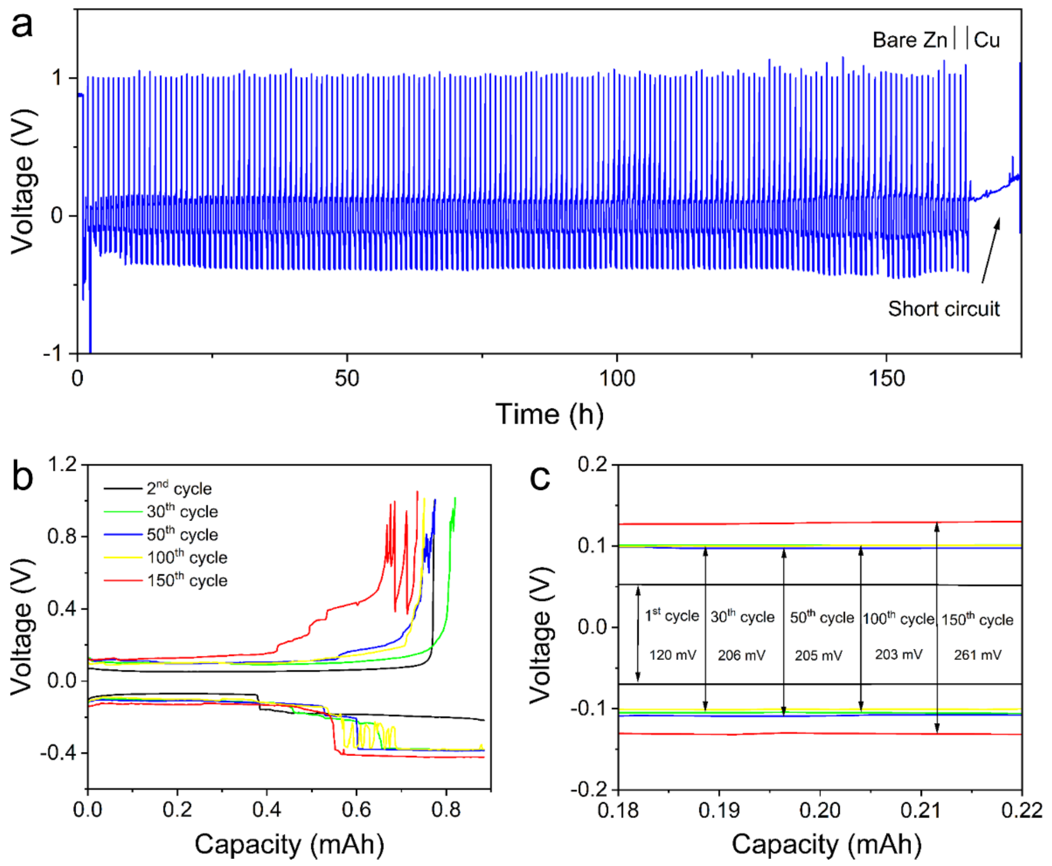

Figure S33. The CE of Zn||Cu half-cell with 3 M $Zn(OTf)_2$ at 1 mA cm^{-2} and 0.5 mAh cm^{-2}. (a) Voltage-time curves, (b) stripping-plating curves at 2nd, 30th, 50th, 100th and 150th cycles and (c) the corresponding polarizations at 1st, 30th, 50th, 100th and 150th cycles.

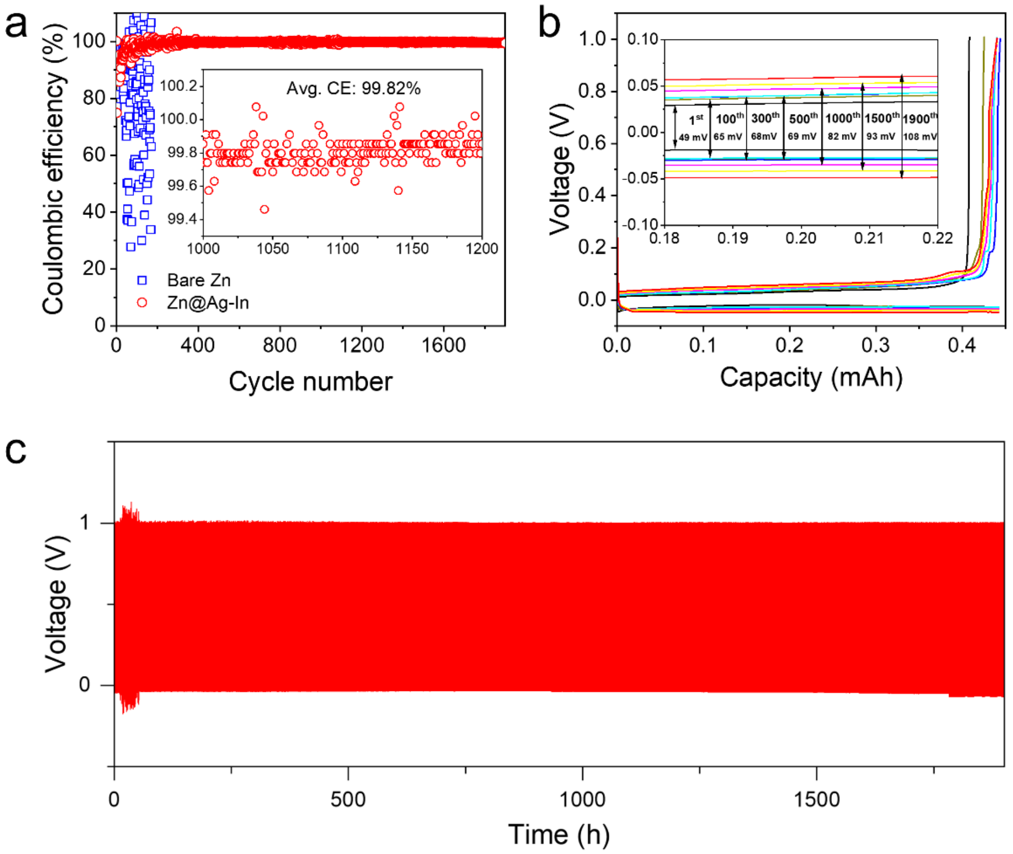

Figure S34. The CE of Zn@Ag-In||Cu half-cell with 3 M $ZnSO_4$ at 1 mA cm^{-2} and 0.5 mAh cm^{-2}. (a) CE curves, (b) stripping-plating curves at 1st, 100th, 300th, 500th, 1000th, 1500th and1900th cycles and (c) the voltage-time curves.

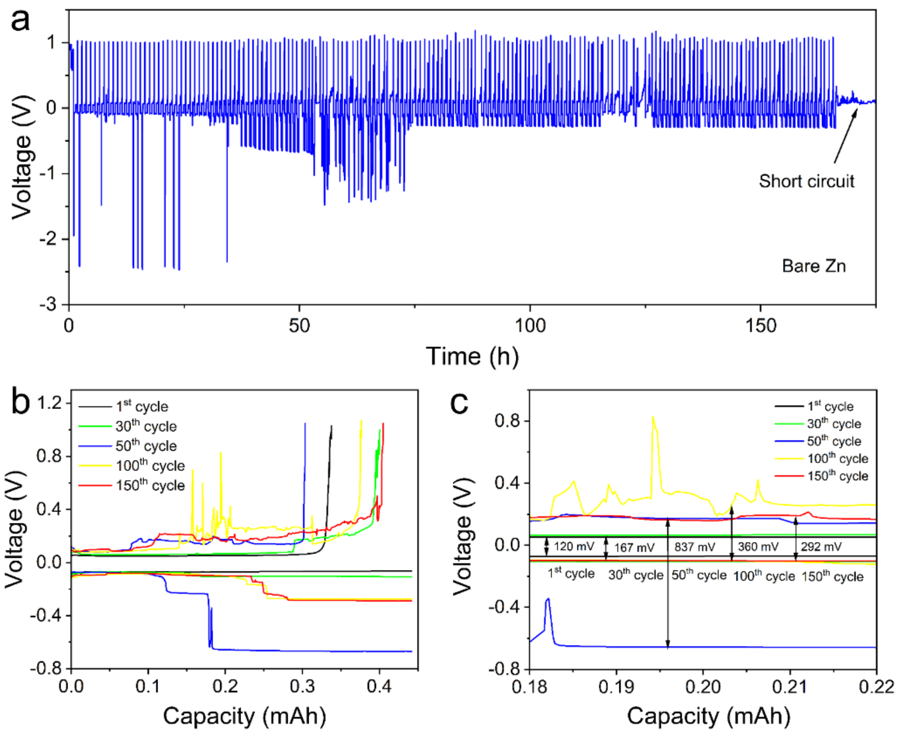

Figure S35. The CE of Zn||Cu half-cell with 3 M $ZnSO_4$ at 1 mA cm^{-2} and 0.5 mAh cm^{-2}. (a) Voltage-time curves, (b) stripping-plating curves at 2nd, 30th, 50th, 100th and 150th cycles and (c) the corresponding polarizations at 1st, 30th, 50th, 100th and 150th cycles.

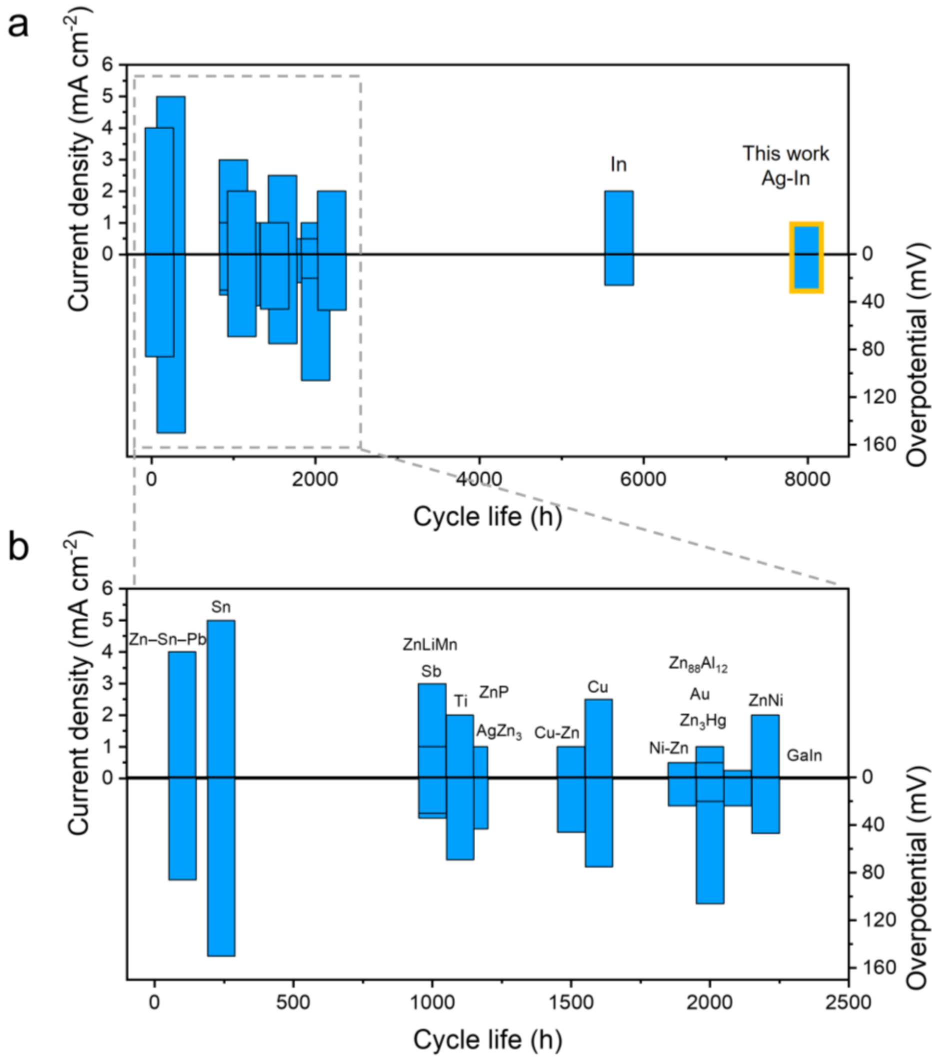

Figure S36. Comparison of cycling performance with other single-heterometallic protective layers.

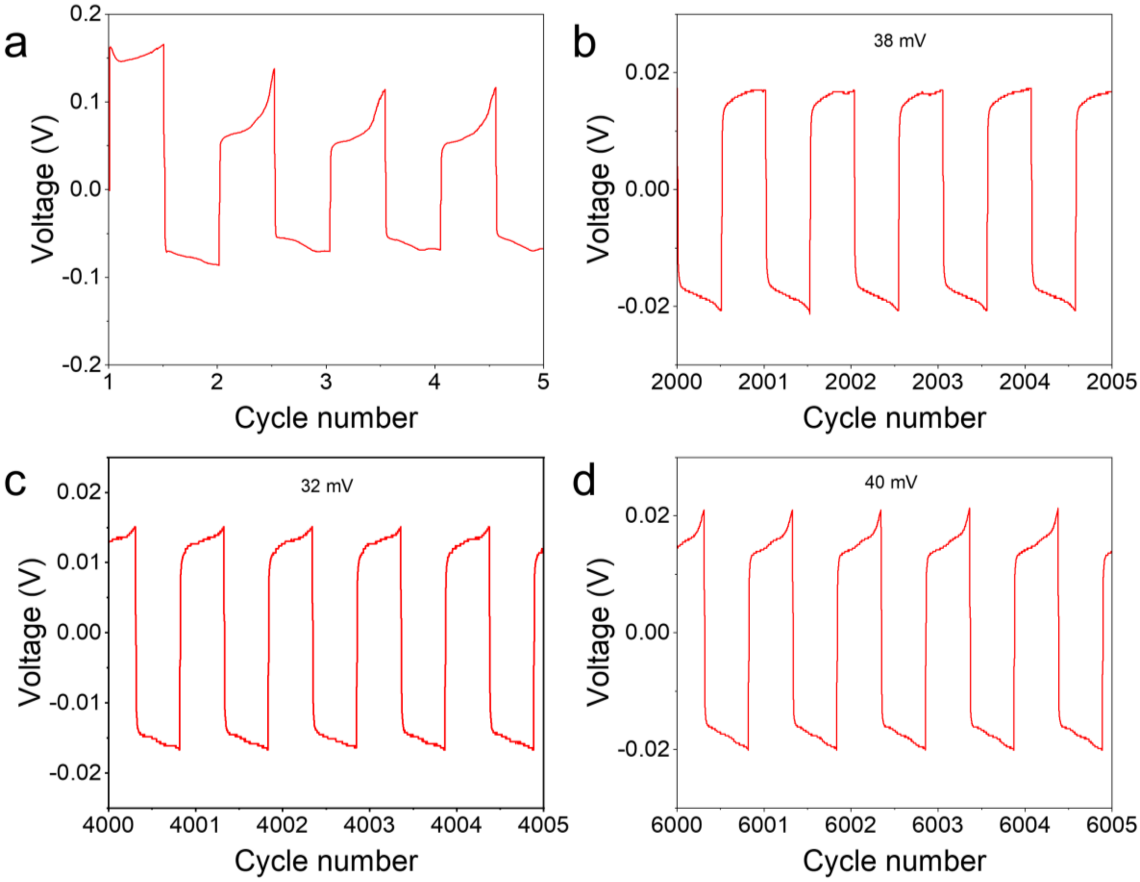

Figure S37. The high resolution curves of Zn@Ag-In||Zn@Ag-In symmetrical cell with a sputting time of 200 s at 1 mA cm^{-2} and 0.5 mAh cm^{-2}.

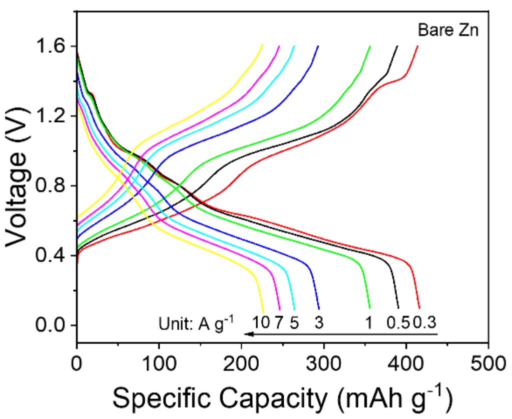

Figure S38. The charge/discharge curves of full cells with bare zinc at different current densities from 0.3 - 10 A g^{-1}.

Supplementary Tables

Table S1. Binding energy (eV) of Zn2p for the Zn@Ag-In electrode after 50 cycles with sputtering times for 0s, 60s and 260s.

**Sputter time (s)**	**$Zn2p_{1/2}$**	**$(Zn\text{-}Ag)2p_{1/2}$**	**$Zn2p_{3/2}$**	**$(Zn\text{-}Ag)2p_{3/2}$**	**Area ratio of $(Zn\text{-}Ag)2p_{3/2}/Zn2p_{3/2}$**
0s	1044.5 eV	1044.0 eV	1021.5 eV	1020.9 eV	0.25
60s	1044.7 eV	1043.8 eV	1021.7 eV	1020.7 eV	0.81
260s	1044.7 eV	1043.8 eV	1021.6 eV	1020.8 eV	3.13

Table S2. Binding energy (eV) of Ag3d for the Zn@Ag-In electrode after 50 cycles with sputtering times for 0s, 60s and 260s.

Sputter time (s)	$Ag3d_{3/2}$	$(Ag-Zn)3d_{3/2}$	$Ag3d_{5/2}$	$(Ag-Zn)3d_{5/2}$	Area ratio of $(Ag-Zn)3d_{5/2}/Ag3d_{5/2}$
0s	373.9 eV	374.5 eV	368.0 eV	368.6 eV	0.71
60s	373.9 eV	375.2 eV	368.0 eV	369.0 eV	0.78
260s	373.7 eV	375.2 eV	367.7 eV	369.2 eV	3.30

Table S3. Binding energy (eV) of In3d for the Zn@Ag-In electrode after 50 cycles with sputtering times for 0s, 60s and 260s.

**Sputter time (s)**	$In3d_{3/2}$	$(In\text{-}O)3d_{3/2}$	$In3d_{5/2}$	$(In\text{-}O)3d_{5/2}$
0s	451.1 eV	452.5 eV	443.6 eV	445.0 eV
60s	451.3 eV	452.0 eV	443.8 eV	444.5 eV
260s	451.2 eV	452.0 eV	443.7 eV	445.0 eV

Table S4. Comparison of symmetric cells cycling performance with other metal/alloy works.

Metallic protective layer	Current density and area capacity	Cyclic life	Voltage hysteresis	References
Ag	0.5 mA cm^{-2}<br>0.5 mAh cm^{-2}	600 h	~ 120 mV	[24]
Ag-AgZn	1 mA cm^{-2}<br>1 mAh cm^{-2}	2100 h	~ 80 mV	[25]
In	2 mA cm^{-2}<br>2 mAh cm^{-2}	5700 h	26 mV	[26]
Sb	3 mA cm^{-2}<br>1 mAh cm^{-2}	1000 h	34 mV	[27]
Sn	5 mA cm^{-2}	240 h	150 mV	[28]
GaIn	0.25 mA cm^{-2}	2100 h	24 mV	[29]
Ni-Zn	0.5 mA cm^{-2}	1900 h	24 mV	[30]
Cu	2.5 mAh cm^{-2}	1600 h	75 mV	[31]
Zn_3Hg	1 mA cm^{-2}<br>1 mAh cm^{-2}	2000 h	8 mV	[32]
Cu-Zn	1 mA cm^{-2}<br>0.5 mAh cm^{-2}	1500 h	46 mV	[33]
Zn-Mn	80 mA cm^{-2}	750 h	/	[34]
Ti	2 mA cm^{-2}<br>2 mAh cm^{-2}	1100 h	27 mV	[35]
Au	0.25 mA cm^{-2}<br>0.05 mAh cm^{-2}	2000 h	106 mV	[36]
$AgZn_3$	1 mA cm^{-2}<br>1 mAh cm^{-2}	1150 h	43.1 mV	[37]
$Zn_{88}Al_{12}$	0.5 mA cm^{-2}	2000 h	20 mV	[38]
ZnLiMn	1 mA cm^{-2}	1000 h	30 mV	[39]
ZnNi	2 mA cm^{-2}<br>1 mAh cm^{-2}	2200 h	47 mV	[40]
ZnP	2 mA cm^{-2}<br>1 mAh cm^{-2}	1100 h	69 mV	[41]

Zn–Sn–Pb	4 mA g^{-1}<br>1 mAh g^{-1}	100 h	86 mV	[42]
**Zn@Ag-In**	**1 mA cm^{-2}**<br>**0.5 mAh cm^{-2}**	**8000 h**	**32 mV**	**This work**

Table S5. Comparison of full cell cycling performance with other works.

Strategies	Full cell	Current density (A g^{-1})	Cycle number	Capacity retention	References
Anode	Zn@$AgZn_3$||VO_2	0.1	2500	50	[37]
Anode	Zn@Ag||CNT/MnO_2	1	400	84.7	[43]
Anode	Zn@In||MnO_2	0.616	300	99	[44]
Anode	Zn@GaIn||MnO_2	1	100	/	[45]
Anode	Zn-Ti||$NH_4V_4O_{10}$	5	4000	85	[46]
Anode	3D Ni-Zn||PANI-V_2O_5·nH_2O	10	1000	80	[47]
Anode	Zn@Cu-Zn||V_2O_5	2	600	88.2	[48]
Anode	Zn@UiO-66-$(COOH)_2$||V_2O_5·nH_2O	1	2400	91	[49]
Anode	Zn@NTP||NaV_3O_8·$1.5H_2O$	5	10,000	/	[50]
Anode	Zn@SIP||$Mg_{0.1}V_2O_5$·H_2O	5	2000	98.3	[51]
Anode	Zn@ZCO||$NH_4V_4O_{10}$	10	2000	100	[52]
Anode	Zn@ZP||V_6O_{13}	2	1000	/	[53]
Anode	Zn@UiO-66||MnO_2	1	50	100	[54]
Electrolyte	HCZE||$LiMn_2O_4$	4C	4000	85	[55]
Electrolyte	$Zn(OTF)_2$ + Me_3EtNOTF||$VOPO_4$	2	6000	88.7	[10]
Electrolyte	ZLT-DMC||$VOPO_4$	2	4300	80	[9]
Electrolyte	$ZnSO_4$+EGME||V_2O_5	0.5	300	/	[56]
Electrolyte	Zeolite-modified||VO_2	2	3000	70.3	[57]
Electrolyte	0.02 M $BMIm^+$||$NH_4V_4O_{10}$	1	1000	/	[58]
Electrolyte	HEE-1.2-3||$NH_4V_4O_{10}$	1	1600	100	[59]
Electrolyte	Quasi-solid||$Na_xV_2O_5$·nH_2O	0.1	50	62.6	[60]
Electrolyte	PCZ-gel||$NH_4V_4O_{10}$	2	2000	86	[61]
Electrolyte	SPS10||V_2O_5·H_2O	1	500	104	[62]

Electrolyte	ImS/ZSO||$NaV_3O_8\cdot1.5H_2O$	20	3000	88	[63]
Electrolyte	67Malt/ZS||$NH_4V_4O_{10}$	0.05	400	84.2	[64]
Electrolyte	Sulfolane-H_2O||$V_2O_5\cdot nH_2O$	5	2000	87	[65]
Electrolyte	P20||$NaV_3O_8\cdot1.5H_2O$	0.2	400	100	[66]
Electrolyte	$ZnSO_4$+$NH_4(H_2PO_4)$||MnO_2	1	1000	/	[67]
Electrolyte	Zn(TFSI)2+Ace||V_2O_5	/	600	99.9	[68]
**Anode**	**Zn@(Ag-In)||ZnVO**	**3**	**10,000**	**90.2**	**This work**

References:

[1] Z. Zheng, X. Zhong, Q. Zhang, M. Zhang, L. Dai, X. Xiao, J. Xu, M. Jiao, B. Wang, H. Li, Y. Jia, R. Mao, G. Zhou, *Nat Commun* **2024**, *15*, 753.

[2] G. Kresse, J. Furthmü, *Efficient Iterative Schemes for Ab Initio Total-Energy Calculations Using a Plane-Wave Basis Set*, **1996**.

[3] J. P. Perdew, K. Burke, M. Ernzerhof, *Generalized Gradient Approximation Made Simple*, **1996**.

[4] S. Grimme, S. Ehrlich, L. Goerigk, *J Comput Chem* **2011**, *32*, 1456–1465.

[5] V. Wang, N. Xu, J. C. Liu, G. Tang, W. T. Geng, *Comput Phys Commun* **2021**, *267*, DOI 10.1016/j.cpc.2021.108033.

[6] G. Henkelman, B. P. Uberuaga, H. Jónsson, *Journal of Chemical Physics* **2000**, *113*, 9901–9904.

[7] K. Momma, F. Izumi, *J Appl Crystallogr* **2008**, *41*, DOI 10.1107/S0021889808012016.

[8] D. Wang, D. Lv, H. Liu, S. Zhang, C. Wang, C. Wang, J. Yang, Y. Qian, *Angewandte Chemie - International Edition* **2022**, *61*, DOI 10.1002/anie.202212839.

[9] H. Jiang, L. Tang, Y. Fu, S. Wang, S. K. Sandstrom, A. M. Scida, G. Li, D. Hoang, J. J. Hong, N. C. Chiu, K. C. Stylianou, W. F. Stickle, D. Wang, J. Li, P. A. Greaney, C. Fang, X. Ji, *Nat Sustain* **2023**, *6*, 806–815.

[10] L. Cao, D. Li, T. Pollard, T. Deng, B. Zhang, C. Yang, L. Chen, J. Vatamanu, E. Hu, M. J. Hourwitz, L. Ma, M. Ding, Q. Li, S. Hou, K. Gaskell, J. T. Fourkas, X. Q. Yang, K. Xu, O. Borodin, C. Wang, *Nat Nanotechnol* **2021**, *16*, 902–910.

[11] F. Wang, O. Borodin, T. Gao, X. Fan, W. Sun, F. Han, A. Faraone, J. A. Dura, K. Xu, C. Wang, *Nat Mater* **2018**, *17*, 543–549.

[12] C. Li, A. Shyamsunder, A. G. Hoane, D. M. Long, C. Y. Kwok, P. G. Kotula, K. R. Zavadil, A. A. Gewirth, L. F. Nazar, *Joule* **2022**, *6*, 1103–1120.

[13] L. Suo, O. Borodin, T. Gao, M. Olguin, J. Ho, X. Fan, C. Luo, C. Wang, K. Xu, *Science (1979)* **2015**, *350*.

[14] H. Qiu, X. Du, J. Zhao, Y. Wang, J. Ju, Z. Chen, Z. Hu, D. Yan, X. Zhou, G. Cui, *Nat Commun* **2019**, *10*, DOI 10.1038/s41467-019-13436-3.

[15] L. Cao, D. Li, E. Hu, J. Xu, T. Deng, L. Ma, Y. Wang, X. Q. Yang, C. Wang, *J Am Chem Soc* **2020**, *142*, 21404–21409.

[16] S. Choudhury, Z. Tu, S. Stalin, D. Vu, K. Fawole, D. Gunceler, R. Sundararaman, L. A. Archer, *Angewandte Chemie* **2017**, *129*, 13250–13257.

[17] E. G. Leggesse, C. L. Chen, J. C. Jiang, *Carbon N Y* **2016**, *103*, 209–216.

[18] H. Jin, S. Xin, C. Chuang, W. Li, H. Wang, J. Zhu, H. Xie, T. Zhang, Y. Wan, Z. Qi, W. Yan, Y.-R. Lu, T.-S. Chan, X. Wu, J. B. Goodenough, H. Ji, X. Duan, *Science (1979)* **2020**, *370*, 192–197.

[19] M. Sek Kim, S. Hun Lee, M.-S. Kim, J.-H. Ryu, K.-R. Lee, L. A. Archer, W. Il Cho, *Sci. Adv.* **2019**, *5*, eaax5587.

[20] W. Zhang, D.-H. Seo, T. Chen, L. Wu, M. Topsakal, Y. Zhu, D. Lu, G. Ceder, F. Wang, *Science (1979)* **2020**, *367*, 1030–1034.

[21] Z. Wang, Q. Su, H. Deng, W. He, J. Lin, Y. Q. Fu, *J Mater Chem A Mater* **2014**, *2*, 13976–13982.

[22] S. Jin, X. Gao, S. Hong, Y. Deng, P. Chen, R. Yang, Y. L. Joo, L. A. Archer, *Joule* **2024**, *8*, 1–8.

[23] J. S. Chang, S. wen Chen, *J Electron Mater* **2015**, *44*, 1134–1143.

[24] X. Zhou, B. Wen, Y. Cai, X. Chen, L. Li, Q. Zhao, S. L. Chou, F. Li, *Angewandte Chemie - International Edition* **2024**, *63*, e202402342.

[25] J. Zheng, X. Liu, Y. Zheng, A. N. Gandi, X. Kuai, Z. Wang, Y. Zhu, Z. Zhuang, H. Liang, *Nano Lett* **2023**, *23*, 6156–6163.

[26] K. Ouyang, D. Ma, N. Zhao, Y. Wang, M. Yang, H. Mi, L. Sun, C. He, P. Zhang, *Adv Funct Mater* **2022**, *32*, 2109749.

[27] L. Hong, L. Y. Wang, Y. Wang, X. Wu, W. Huang, Y. Zhou, K. X. Wang, J. S. Chen, *Advanced Science* **2022**, *9*, 2104866.

[28] L. Wang, W. Huang, W. Guo, Z. H. Guo, C. Chang, L. Gao, X. Pu, *Adv Funct Mater* **2022**, *32*, 2108533.

[29] C. Liu, Z. Luo, W. Deng, W. Wei, L. Chen, A. Pan, J. Ma, C. Wang, L. Zhu, L. Xie, X. Y. Cao, J. Hu, G. Zou, H. Hou, X. Ji, *ACS Energy Lett* **2021**, *6*, 675–683.

[30] Q. Zhang, Y. Dai, K. Zhao, C. Zhang, R. Lu, J. Li, S. Jin, L. Zhang, Q. An, L. Mai, *Nano Res* **2023**, *16*, 11604–11611.

[31] L. Zhou, F. Yang, S. Zeng, X. Gao, X. Liu, X. Cao, P. Yu, X. Lu, *Adv Funct Mater* **2022**, *32*, 2110829.

[32] H. Tao, Z. Hou, L. Zhang, X. Yang, L. Z. Fan, *Chemical Engineering Journal* **2022**, *450*, 138048.

[33] Z. Cai, Y. Ou, J. Wang, R. Xiao, L. Fu, Z. Yuan, R. Zhan, Y. Sun, *Energy Storage Mater* **2020**, *27*, 205–211.

[34] H. Tian, Z. Li, G. Feng, Z. Yang, D. Fox, M. Wang, H. Zhou, L. Zhai, A. Kushima, Y. Du, Z. Feng, X. Shan, Y. Yang, *Nat Commun* **2021**, *12*, 237.

[35] Y. Zhao, S. Guo, M. Chen, B. Lu, X. Zhang, S. Liang, J. Zhou, *Nat Commun* **2023**, *14*, 7080.

[36] M. Cui, Y. Xiao, L. Kang, W. Du, Y. Gao, X. Sun, Y. Zhou, X. Li, H. Li, F. Jiang, C. Zhi, *ACS Appl Energy Mater* **2019**, *2*, 6490–6496.

[37] H. Lu, Q. Jin, X. Jiang, Z. M. Dang, D. Zhang, Y. Jin, *Small* **2022**, *18*, 2200131.

[38] S. B. Wang, Q. Ran, R. Q. Yao, H. Shi, Z. Wen, M. Zhao, X. Y. Lang, Q. Jiang, *Nat Commun* **2020**, *11*, 1634.

[39] Y. Zhang, X. Yang, Y. Hu, K. Hu, X. Lin, X. Liu, K. M. Reddy, G. Xie, H. J. Qiu, *Small* **2022**, *18*, 2200787.

[40] P. Cao, J. Tang, A. Wei, Q. Bai, Q. Meng, S. Fan, H. Ye, Y. Zhou, X. Zhou, J. Yang, *ACS Appl Mater Interfaces* **2021**, *13*, 48855–48864.

[41] T. Wang, Q. Xi, Y. Li, H. Fu, Y. Hua, E. G. Shankar, A. K. Kakarla, J. S. Yu, *Advanced Science* **2022**, *9*, 2200155.

[42] X. Fan, H. Yang, X. Wang, J. Han, Y. Wu, L. Gou, D. L. Li, Y. L. Ding, *Adv Mater Interfaces* **2021**, *8*, 2002184.

[43] Y. Wang, Y. Chen, W. Liu, X. Ni, P. Qing, Q. Zhao, W. Wei, X. Ji, J. Ma, L. Chen, *J Mater Chem A Mater* **2021**, *9*, 8452–8461.

[44] P. Xiao, H. Li, J. Fu, C. Zeng, Y. Zhao, T. Zhai, H. Li, *Energy Environ Sci* **2022**, *15*, 1638–1646.

[45] C. Liu, Z. Luo, W. Deng, W. Wei, L. Chen, A. Pan, J. Ma, C. Wang, L. Zhu, L. Xie, X. Y. Cao, J. Hu, G. Zou, H. Hou, X. Ji, *ACS Energy Lett* **2021**, *6*, 675–683.

[46] Y. Zhao, S. Guo, M. Chen, B. Lu, X. Zhang, S. Liang, J. Zhou, *Nat Commun* **2023**, *14*, 7080.

[47] G. Zhang, X. Zhang, H. Liu, J. Li, Y. Chen, H. Duan, *Adv Energy Mater* **2021**, *11*, DOI 10.1002/aenm.202003927.

[48] B. Li, K. Yang, J. Ma, P. Shi, L. Chen, C. Chen, X. Hong, X. Cheng, M. C. Tang, Y. B. He, F. Kang, *Angewandte Chemie - International Edition* **2022**, *61*, DOI 10.1002/anie.202212587.

[49] W. L. Xin, J. Xiao, J. Li, L. Zhang, H. Peng, Z. Yan, Z. Zhu, *Energy Storage Mater* **2023**, *56*, 76–86.

[50] M. Zhao, Y. Lv, S. Zhao, Y. Xiao, J. Niu, Q. Yang, J. Qiu, F. Wang, S. Chen, *Advanced Materials* **2022**, *34*, DOI 10.1002/adma.202206239.

[51] M. Zhao, J. Rong, F. Huo, Y. Lv, B. Yue, Y. Xiao, Y. Chen, G. Hou, J. Qiu, S. Chen, *Advanced Materials* **2022**, *34*, DOI 10.1002/adma.202203153.

[52] P. Wang, S. Liang, C. Chen, X. Xie, J. Chen, Z. Liu, Y. Tang, B. Lu, J. Zhou, *Advanced Materials* **2022**, *34*, DOI 10.1002/adma.202202733.

[53] Z. Xing, Y. Sun, X. Xie, Y. Tang, G. Xu, J. Han, B. Lu, S. Liang, G. Chen, J. Zhou, *Angewandte Chemie - International Edition* **2023**, *62*, DOI 10.1002/anie.202215324.

[54] M. Liu, L. Yang, H. Liu, A. Amine, Q. Zhao, Y. Song, J. Yang, K. Wang, F. Pan, *ACS Appl Mater Interfaces* **2019**, *11*, 32046–32051.

[55] F. Wang, O. Borodin, T. Gao, X. Fan, W. Sun, F. Han, A. Faraone, J. A. Dura, K. Xu, C. Wang, *Nat Mater* **2018**, *17*, 543–549.

[56] M. Liu, L. Yao, Y. Ji, M. Zhang, Y. Gan, Y. Cai, H. Li, W. Zhao, Y. Zhao, Z. Zou, R. Qin, Y. Wang, L. Liu, H. Liu, K. Yang, T. S. Miller, F. Pan, J. Yang, *Nano Lett* **2023**, *23*, 541–549.

[57] H. Yang, Y. Qiao, Z. Chang, H. Deng, X. Zhu, R. Zhu, Z. Xiong, P. He, H. Zhou, *Advanced Materials* **2021**, *33*, DOI 10.1002/adma.202102415.

[58] H. Zhang, Y. Zhong, J. Li, Y. Liao, J. Zeng, Y. Shen, L. Yuan, Z. Li, Y. Huang, *Adv Energy Mater* **2023**, *13*, DOI 10.1002/aenm.202203254.

[59] M. Han, J. Huang, X. Xie, T. C. Li, J. Huang, S. Liang, J. Zhou, H. J. Fan, *Adv Funct Mater* **2022**, *32*, DOI 10.1002/adfm.202110957.

[60] S. Guo, L. Qin, C. Hu, L. Li, Z. Luo, G. Fang, S. Liang, *Adv Energy Mater* **2022**, *12*, DOI 10.1002/aenm.202200730.

[61] H. Zhang, X. Gan, Z. Song, J. Zhou, *Angewandte Chemie - International Edition* **2023**, *62*, DOI 10.1002/anie.202217833.

[62] Y. Lin, Z. Mai, H. Liang, Y. Li, G. Yang, C. Wang, *Energy Environ Sci* **2023**, *16*, 687–697.

[63] Y. Lv, M. Zhao, Y. Du, Y. Kang, Y. Xiao, S. Chen, *Energy Environ Sci* **2022**, *15*, 4748–4760.

[64] W. Chen, S. Guo, L. Qin, L. Li, X. Cao, J. Zhou, Z. Luo, G. Fang, S. Liang, *Adv Funct Mater* **2022**, *32*, DOI 10.1002/adfm.202112609.

[65] M. Li, X. Wang, J. Hu, J. Zhu, C. Niu, H. Zhang, C. Li, B. Wu, C. Han, L. Mai, *Angewandte Chemie - International Edition* **2023**, *62*, DOI 10.1002/anie.202215552.

[66] D. S. Liu, Y. Zhang, S. Liu, L. Wei, S. You, D. Chen, M. Ye, Y. Yang, X. Rui, Y. Qin, C. C. Li, *Adv Funct Mater* **2022**, *32*, DOI 10.1002/adfm.202111714.

[67] W. Zhang, Y. Dai, R. Chen, Z. Xu, J. Li, W. Zong, H. Li, Z. Li, Z. Zhang, J. Zhu, F. Guo, X. Gao, Z. Du, J. Chen, T. Wang, G. He, I. P. Parkin, *Angewandte Chemie - International Edition* **2023**, *62*, e202212695.

[68] H. Qiu, X. Du, J. Zhao, Y. Wang, J. Ju, Z. Chen, Z. Hu, D. Yan, X. Zhou, G. Cui, *Nat Commun* **2019**, *10*, 5374.
